# Integrative Differential Expression Analysis for Multiple EXperiments (IDEAMEX): A Web Server Tool for Integrated RNA-Seq Data Analysis

**DOI:** 10.3389/fgene.2019.00279

**Published:** 2019-03-29

**Authors:** Verónica Jiménez-Jacinto, Alejandro Sanchez-Flores, Leticia Vega-Alvarado

**Affiliations:** ^1^Unidad Universitaria de Secuenciación Masiva y Bioinformática, Instituto de Biotecnología, Universidad Nacional Autónoma de México, Cuernavaca, Mexico; ^2^Instituto de Ciencias Aplicadas y Tecnología, Universidad Nacional Autónoma de México, Mexico City, Mexico

**Keywords:** bioinformatics, RNA-Seq, differential expression, NGS, transcriptomics

## Abstract

The current DNA sequencing technologies and their high-throughput yield, allowed the thrive of genomic and transcriptomic experiments but it also have generated big data problem. Due to this exponential growth of sequencing data, also the complexity of managing, processing and interpreting it in order to generate results, has raised. Therefore, the demand of easy-to-use friendly software and websites to run bioinformatic tools is imminent. In particular, RNA-Seq and differential expression analysis have become a popular and useful method to evaluate the genetic expression change in any organism. However, many scientists struggle with the data analysis since most of the available tools are implemented in a UNIX-based environment. Therefore, we have developed the web server IDEAMEX (Integrative Differential Expression Analysis for Multiple EXperiments). The IDEAMEX pipeline needs a raw count table for as many desired replicates and conditions, allowing the user to select which conditions will be compared, instead of doing all-vs.-all comparisons. The whole process consists of three main steps (1) Data Analysis: that allows a preliminary analysis for quality control based on the data distribution per sample, using different types of graphs; (2) Differential expression: performs the differential expression analysis with or without batch effect error awareness, using the bioconductor packages, NOISeq, limma-Voom, DESeq2 and edgeR, and generate reports for each method; (3) Result integration: the obtained results the integrated results are reported using different graphical outputs such as correlograms, heatmaps, Venn diagrams and text lists. Our server allows an easy and friendly visualization for results, providing an easy interaction during the analysis process, as well as error tracking and debugging by providing output log files. The server is currently available and can be accessed at http://www.uusmb.unam.mx/ideamex/ where the documentation and example input files are provided. We consider that this web server can help other researchers with no previous bioinformatic knowledge, to perform their analyses in a simple manner.

## Introduction

Transcriptomics experiments have been used widely to measure the RNA levels expressed in tissues or cells from practically any organism. This approach has been used since the implementation of Northern blots hybridization analysis and was scaled up by the development of microarray technology. However, transcriptomics has been improved with the aid of sequencing technologies which recently have been replacing microarrays by using RNA sequencing (RNA-Seq) experiments to evaluate gene expression at a genome-wide scale. Therefore, either microarrays or RNA-Seq technologies have generated a massive amount of data results that demands *ad hoc* methods to fully analyze and compare gene expression between different conditions, tissues or cell populations for a given organism.

To quantify the transcription levels and identify differential expressed genes under different conditions, using RNA-Seq data from high-throughput sequencing technologies, a general workflow can be described: (1) quality control of RNA-Seq reads (Babraham Bioinformatics - FastQC A Quality Control tool for High Throughput Sequence Data Babraham Bioinformatics - FastQC A Quality Control tool for High Throughput Sequence Data); (2) read trimming or filtering (Chen et al., 2017; Roser et al., 2018); (3) mapping trimmed/filtered reads to a reference (genome or transcriptome) (Li and Durbin, 2009; Langmead and Salzberg, 2012; Kim et al., 2013; Wu et al., 2016); (4) obtaining the read count for each gene (Quinlan and Hall, 2010; Li and Dewey, 2011; Roberts et al., 2011) and (5) differential expression analysis (Anders and Huber, 2010; Tarazona et al., 2011; McCarthy et al., 2012; Love et al., 2014; Ritchie et al., 2015). Currently, due to the size of datasets, steps 1 to 4 have to be performed by the user and many tools for each step are available and have been widely used and cited elsewhere. However, the differential expression analysis is probably the most important step that allows the user to interpret the biological information regarding the expression profiles of a given organism under different conditions.

The gene expression profile contains the information regarding genes related to the organism response to a certain condition. To retrieve such information, the differential expression analysis has to be performed and it requires statistical methods to differentiate between expression changes due to the tested conditions and biological “noise” or variability. Currently, several computational tools have been developed mainly in the programming language R and packages are available at the Bioconductor project repository (Huber et al., 2015). However, R language and packages have to be used mainly through a UNIX-based operating system and by command-line instructions which requires a certain level of programming skills. Therefore, non-bioinformatics researchers demand either a Graphical User Interface (GUI) in order to use differential expression tools or web-based applications. A GUI-based solution still requires a local installation of all packages needed for the differential expression analysis and this could remain challenging. The web-based applications are now emerging (de Jong et al., 2015; Monier et al., 2018; Zhang et al., 2018) as friendlier option to perform the differential expression analysis in a more friendly way and without installing software in a local computer.

Here, we introduce the IDEAMEX web server (Integrative Differential Expression Analysis for Multiple EXperiments) that uses as input an RNA-Seq raw count table in text format and generates results using bioconductor packages NOISeq, limma-voom, DESeq2 and edgeR. These packages have been constanlty benchmarked and presented the most reliable results with different datasets and gold-standards (Seyednasrollah et al., 2015; Costa-Silva et al., 2017). In this work, we demonstrate the functionality of IDEAMEX, using RNA-Seq data from a previous publication (Olvera et al., 2017) where the differential expression analysis in tilapia liver was performed, in addition to other datasets used as examples to test the website.

Our server has been used in several projects and has been visited from different world-wide locations as recorded in our site tracker. IDEAMEX is available and can be accessed at http://www.uusmb.unam.mx/ideamex/ where the documentation and example input files are provided. Our server offers a web server-based analysis that can help researchers with no previous bioinformatic knowledge, to perform their transcriptomic analyses in a simple manner, in order to interpret the biological data contained in their RNA-Seq experiments.

## Materials and Methods

### Web Server Description

The web page is hosted by the “Unidad Universitaria de Secuenciación Masiva y Bioinformática” core lab facility, at the “Instituto de Biotecnología” of the “Universidad Nacional Autónoma de México, Campus Morelos located in Cuernavaca, Morelos, México.” A Linux box computer with Ubuntu 14.04 LTS with the following hardware main characteristics: Intel Core i7 4770 processor; 32 Gbytes of DDR3 RAM and 1 Tbyte of hard disk storage.

The deployment was implemented using the Apache HTTP server version 2.4.7 with a PHP v5.5.9 front-end that coordinates the writing of the input and output files to a SQL database through a POSGRES Relational Data Base Manager (RDBM) server (psql version 9.3.22. The installed R version is 3.5.2. The web server can be accessed at http://www.uusmb.unam.mx/ideamex/.

The web server interface has been tested using different web browsers and different operative systems. Using Microsoft Windows 10: Microsoft EdgeHTML 17.17134; Google Chrome version 72.0.3626.109 (Official Build) (64-bit); Mozilla Firefox Quantum 63.0 (64-bit). Using MacOS Sierra 10.13.6: Safari 12.0.3; Google Chrome 71.0.3578.98 (64-bit). Using Linux Ubuntu 16.04 LTS: Mozilla Firefox Quantum 65.0.

Additionally, the scripts and binaries used in the web server can be found in the public repository https://github.com/leticiaVega/IDEAMEX

### RNA-Seq Examples and Data From Tilapia Liver Experiment

We used as example to test our website two datasets. The first example contains data from the Pasilla Bioconductor library (Brooks et al., 2010), taking in account only the gene level counts. This dataset contains RNA-Seq count data for treated and untreated cells from the S2-DRSC cell line. The second example file which can be used to test the batch effect error awareness, was taken from the NBPSeq CRAN package (Di et al., 2014). This dataset contains the *Arabidopsis thaliana* RNA-Seq data (Cumbie et al., 2011), comparing ΔhrcC challenged and mock-inoculated samples. In this case, the samples were collected in three batches.

We also obtained RNA-Seq publicly available data already reported (Olvera et al., 2017) that was generated to determine the effect of 3,5-di-iodothyronine (T2) and 3,5,3′-tri-iodothyronine (T3) exogenous treatment on the transcriptome of tilapia (Oreochromis niloticus) liver. For control and each hormone treatment, two biological replicates were generated. The FASTQ raw data can be found under the following SRA identifiers: SRX2630485, SRX2630486, SRX2630487, SRX2630488, SRX2630489, and SRX2630490.

Briefly, the quality control(QC) and filtering for the raw data was performed using the FASTQC software (Babraham Bioinformatics - FastQC A Quality Control tool for High Throughput Sequence Data Babraham Bioinformatics - FastQC A Quality Control tool for High Throughput Sequence Data) and contamination and adapter removal was carried out using in-house Perl scripts. QC’ed reads were mapped using the Bowtie 1.1.234 aligner (Langmead et al., 2009) to the annotated *Oreochromis_niloticus* (Orenil1.0.cds.all, 21,437 coding genes) CDS dataset downloaded from Ensembl repository database (Aken et al., 2016) using the BioMart utility. Quantification and repetitiveness normalization were carried out using eXpress software 1.535 (Roberts et al., 2011). Total effective counts for each sample were merged; a matrix was generated using the “abundance_estimates_to_matrix.pl” Perl script included in the Trinity pipeline (Grabherr et al., 2011; Roberts et al., 2011). The resulting matrix was used as input for the differential expression analysis in the IDEAMEX web server. The select parameters were: *p*-adj/FDR = 0.05; logFC = 2; CPM = 1.

### Differential Expression Packages

Based on the parameters defined by the user, 4 different R (version 3.5.2) packages for differential expression analysis are run: edgeR version 3.24.3 (Anders and Huber, 2010), using TMM normalization method (works with or without replicates); limma-Voom version 3.38.3 (Ritchie et al., 2015), using log2-counts per million normalization method (works with replicates only); DESeq2 version 1.22.2 (Love et al., 2014), with DESeq2-default normalization method (works with or without replicates) and NOISeq version 2.26.1 (Tarazona et al., 2011), with TMM normalization method (works with or without replicates). Other packages used in the server are: VennDiagram 1.6.20; ggplot2 3.1.0; UpSetR 1.3.3; corrplot 0.84 and ComplexHeatmap 1.20.0. The packages can change depending on the R programming language version, but all changes are reported to the user in log files that contain all details about the commands and parameters used for the analysis.

## Results

### The IDEAMEX Web Server Implementation

**Figure 1:**
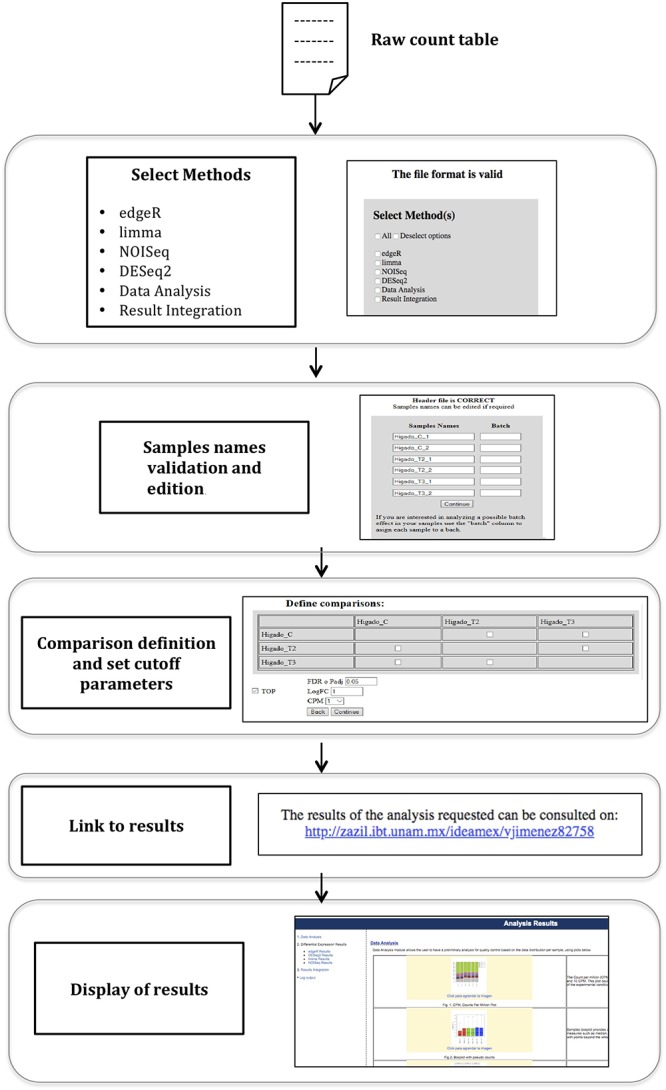
IDEAMEX workflow diagram. The web server workflow starts with the loading and validation of the raw count table as input. Then, the user selects one or more methods for differential expression analysis, data analysis and results integration. An optional step to edit the sample names is available. The user designs the comparison matrix by selecting which conditions will be compared. A link to the results is generated and after a few minutes, the results are presented in the Analysis Results web page.

The general workflow used in the IDEAMEX web server can be observed in Figure 1. First, the user has to enter a valid email address that will be used to report the follow up or the differential expression analysis to the user. In a nutshell, the pipeline starts with a raw count table for as many desired replicates and conditions, allowing the user to select which conditions will be compared, instead of doing all-vs.-all comparisons. After the web server validates the input format, the user can edit the sample names select one or more differential expression methods and the parameters to filter results. Additionally, the user can indicate if the samples belong to different batches so the selected differential expression methods, can correct any possible batch effect Then, the data analysis step is performed where a preliminary quality control report is generated, based on the data distribution per sample. Next, the differential expression analysis is performed using one or more selected methods. Finally, the result from the different selected methods are integrated and are reported using Venn diagrams, a upset bar plot graph and text files for further filtering and analysis. Several additional plots are generated including correlograms to check the consistency between some calculations and heatmaps. Further details and study cases for dataset examples are described in the IDEAMEX User Manual that can be downloaded from the website. To demonstrate the functionality of our web server, we used a dataset generated from an RNA-Seq experiment to compare the effect of thyroid hormones in tilapia liver (see Materials and Methods).

Optionally, the user can perform a full registration at the IDEAMEX homepage, in order to keep track of all projects results. The sample name format should have a suffix_[0-9] structure: nameCond1_1, nameCond1_2, … , nameCond1_n, nameCond2_1, nameCond2_2, … , nameCond2_m. Once the input file is validated, the server can infer the replicates from the suffix before the underscore symbol and the replicate number will be the digit after the underscore symbol. However, during the input loading, the user can edit these names. In case of samples being prepared in different batches, this information can be specified in the same window the sample names are edited. Indicating samples in different batches will turn on the batch effect error correction of different methods. Importantly, use this option only if you have knowledge of samples from a given condition, being prepared in a different batch which can give the experiment an extra variability. The user manual has a case of study for samples with batch effect.

In this work, the samples were named liverC_1, liverC_2 for replicates of control condition (no treatment) and liverT2_1, liverT2_2, liverT3_1, liverT3_2 for replicates that correspond to each of the 3,5-T2 and 3’,3,5-T3 (T2 and T3) thyroid hormones treatments. A raw count table (Supplementary Material S1) in tab-separated text format, was generated and fed to the IDEAMEX web server. A snipped of the input raw count table is shown in Table 1.

**Table 1:** Raw count table example.

	LiverC_1	LiverC_2	LiverT2_1	LiverT2_2	LiverT3_1	LiverT3_2
ENSONIT00000002512	6.816486	5.866294	11.949044	7.285873	14.838847	7.979772
ENSONIT00000002995	0.000000	0.000000	0.001585	0.009734	0.000334	0.752950
ENSONIT00000006143	33.849657	109.674115	127.148250	141.191874	181.345619	132.397050
ENSONIT00000026691	0.000000	0.000000	0.000000	0.000000	0.000000	0.000000
ENSONIT00000008087	74.458461	359.525580	149.166187	161.170914	235.990094	237.782394
ENSONIT00000021608	59.602367	101.722543	255.731580	259.076778	364.441300	329.630108
ENSONIT00000008926	0.000000	8.473091	33.032248	28.360464	21.724295	14.028806
ENSONIT00000011237	0.000000	0.000000	0.000000	0.000000	0.000000	0.000000
ENSONIT00000021761	59.306830	135.526032	162.356881	113.464849	238.652733	233.360459

*The sample names denote the condition naming and replicate number. liverC_N, RNA-Seq counts for liver tissue with no treatment. liverT2_N, RNA-Seq counts for liver tissue with T2 hormone treatment. liverT3_N, RNA-Seq counts for liver tissue with T3 hormone treatment. N, replicate number. Raw count table should be in simple text format*.

### Input and Data Quality Control

The next step is to select the differential analysis method(s), the data quality analysis and the result integration by clicking on each box. It is recommended to click on the “select all” box to perform a full analysis. Afterward, the cut-off values for statistical confidence (*p*-adj and False Discovery Rate [FDR]), normalization (CPM) and transcript abundance difference (logFC) can be selected. Also, the comparison matrix can be defined to establish which samples or conditions will be compared.

A link to the Analysis Results web page will be generated, where the user results can find a link to the “(1) Data Analysis” section. A series of plots are displayed, allowing the user to have a preliminary analysis for quality control based on the data distribution per sample. All conditions defined in the raw count table are depicted as boxplots, CPM bar plots, density plots, principal components analysis (PCA) plots and multi-dimensional scaling (MDS) plots. Inspection and evaluation of these plots are essential steps for the interpretation of the differential expression analysis.

#### CPM Plot Evaluation

In gene expression analysis, only a fraction of genes is expected to show differential expression between experimental conditions. The Count per million (CPM) plot shows the number of genes within each sample, having no counts (CPM = 0) or more than 1, 2, 5, or 10 CPM. This plot could help the user to decide the threshold to remove very low expressed genes in any of the experimental conditions. The default CPM cut-off value of 1 can be changed according to the user judgment, but it has to be done by re-running the analysis.

**Figure 2:**
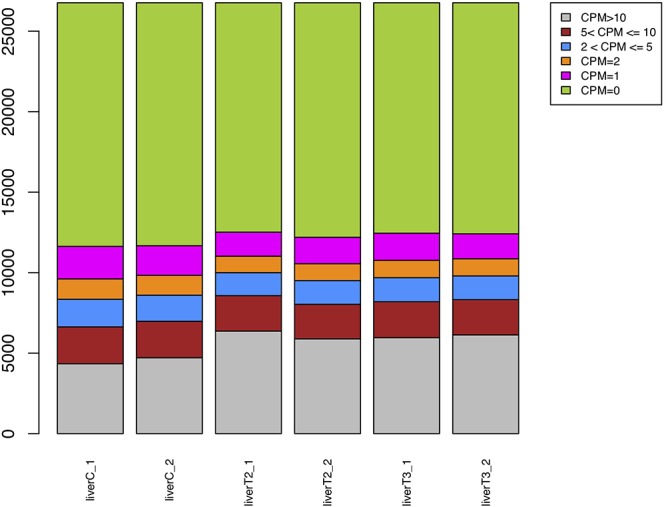
CPM plot. A bar plot for each sample is generated where Counts Per Million for each gene are represented.

As observed in Figure 2, there is an increase of genes with CPM > 10 in the T2 and T3 samples, compared to the C condition. Also, the group of genes with CPM = 2 were decreased in T2 and T3 compared to the C condition. Approximately, ∼70% of the genes presented no counts. This plot is the first glance to the expression profile for the compared conditions. For this particular case, CPM = 1 is a convenient cut-off value which was the default option.

#### Boxplot Evaluation

**Figure 3:**
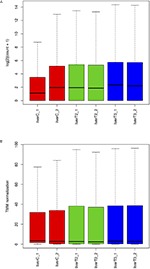
Boxplot with normalized counts. The frequency distribution and some statistics like mean, median and outliers are represented in these plots. **(A)** log2 normalized counts. **(B)** TMM normalized counts.

Figure 3 presents the boxplots which provide an easy way to visualize the count distribution in each sample. If the count values distribution is highly skewed, then data transformation can be applied to roughly normalize the distribution. Figure 3A presents the log2 normalized data (pseudo-counts) and Figure 3B depicts the normalized data using the Trimmed Mean of *M*-values (TMM) method which is used for the differential expression analysis in edgeR and NOIseq packages. As observed, TMM normalization adjust the data according to the sequencing yield of each sample. The boxplot is an easy way to visualize the data distribution since it shows statistical measures such as median, quartiles, minimum and maximum values. Whiskers are also drawn extending beyond each end of the box with points beyond the whiskers typically indicating count outliers. In the log2 boxplot, the sequencing yield difference per sample is very evident. In this case, the control samples have fewer reads than the other samples. However, TMM normalization can fix this problem and this is why several differential expression methods have implemented this normalization procedure.

It is important to mention that the user will find a pair of boxplots, PCA and MDS graphs, since the data is plotted using pseudo-counts and TMM values.

#### Density Plot Evaluation

**Figure 4:**
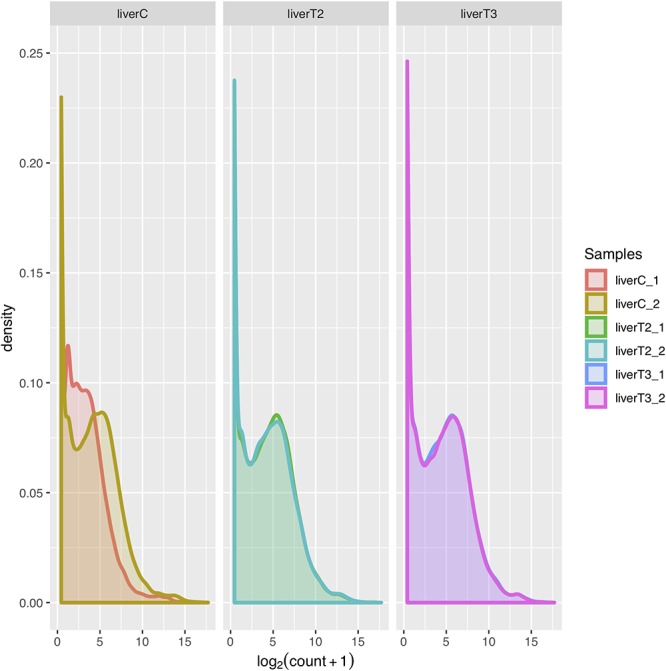
Density plots. The count distribution between replicates and conditions.

The normalized count distributions can also be summarized by means of a density plot. Density plot provide more detail by enabling the detection of a dissimilarity in replicate count distribution. Ideally, the density plot for each replicate for a given condition, should greatly overlap indicating lower variability between replicates. Figure 4 shows a density plot for the samples where replicates for the C condition, indicating certain dissimilarity in replicates for that condition.

#### PCA Plot Evaluation

**Figure 5:**
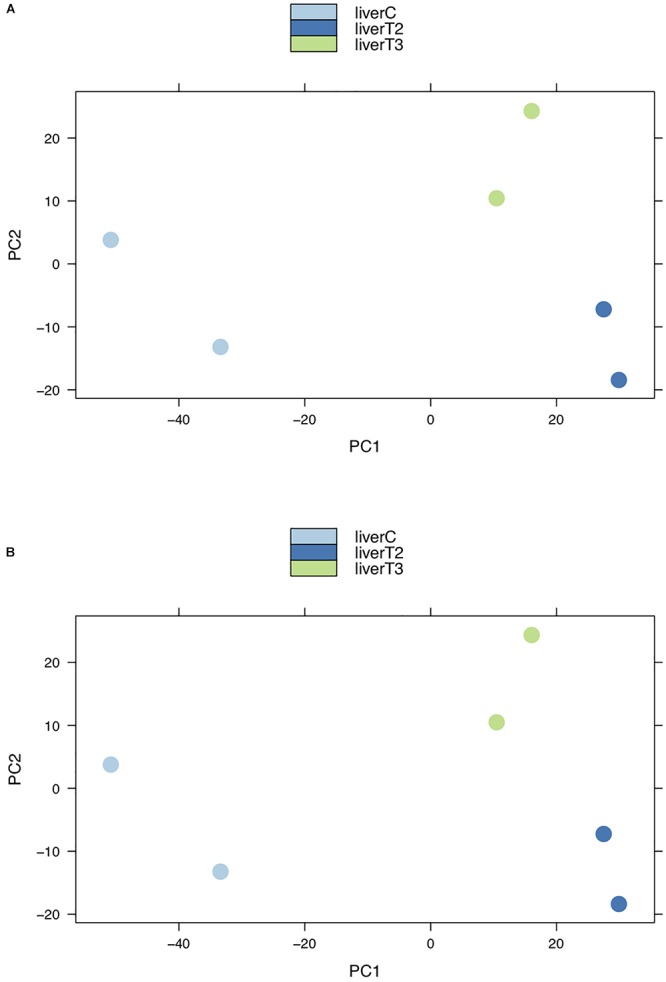
PCA plots. Groups of samples can be analyzed using Principal Component Analysis (PCA) plots where replicates of a certain conditions are clustered together. Clusters from different conditions are separated. **(A)** log2 normalized counts. **(B)** TMM normalized counts.

This type of plot is useful for visualizing the overall effect of experimental covariates and batch effects. In the context of RNA-Seq analysis, PCA shows groups of samples that ideally will correspond to each condition. Clustering first by the most significant group, then by progressively less significant groups. Figure 5 depicts how the 3 conditions (C, T2, and T3) form separate clusters, although some dispersion between replicates can be observed. This suggest that the variability among individuals was high, but due to the cluster separation it shouldn’t affect the analysis. When a replicate is grouped with other samples from different conditions, is recommended to removed it from the analysis if there are enough replicates left (at least two). Also, this plot could indicate if there is a batch effect problem, where samples in a same condition are very disperse in the plot. In that case, the user can rerun the analysis indicating which samples could belong to a different batch. However, we recommend to confirm this with records from the preparation of the samples in the wet lab.

#### MDS Plot Evaluation

**Figure 6:**
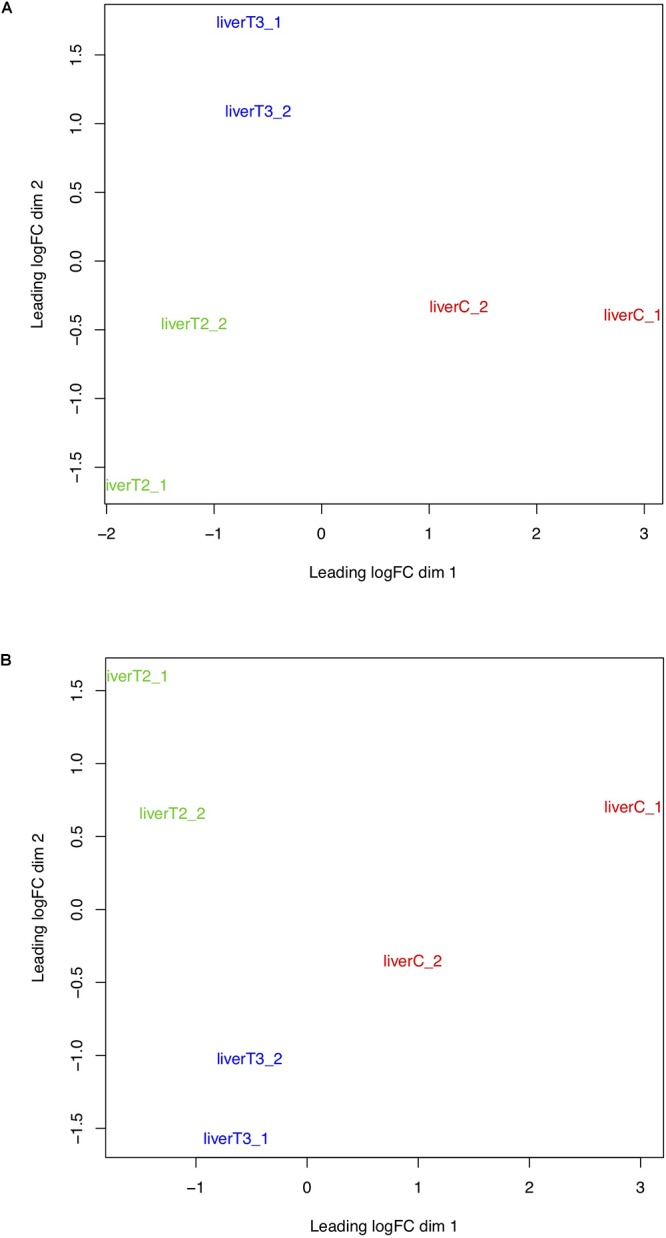
MDS plots. Groups of samples can be analyzed using Multi-dimensional scaling (MDS) plots where the distance between samples and conditions reflect their similarity. **(A)** log2 normalized counts. **(B)** TMM normalized counts.

Multi-dimensional scaling (MDS) is a technique that is used to create a visual representation of the pattern of proximities (similarities, dissimilarities, or distances) among a set of objects. In the context of RNA-Seq analysis, MDS plot shows variation among RNA-Seq samples, the more is the distance between sample, the higher is their dissimilarity. Therefore, samples belonging to the same condition or treatment should be closer to each other and distant to other conditions. However, if different conditions are grouped together, this could mean that those treatments or conditions have a very similar effect. Worst-case scenario, the user can suspect of a sample mislabeling. Conceptually, MDS and PCA plots can provide the same information and as observed in Figure 6, samples belonging to C, T2, and T3 form separate clusters with a certain dispersion among replicates. Similarly, to the PCA plot, this plot could indicate if there is a batch effect problem, where samples in a same condition are very disperse in the plot. Again, we recommend to confirm the preparation of the samples, by checking records from the preparation of the samples in the wet lab.

### Differential Expression Analysis

The “(2) Differential Expression Results” section has links with the name of each selected method, where the user can display the analysis output. A detailed description of each method output can be found in the User Manual at the IDEAMEX web page. However, here we describe the generated graphs for a better interpretation. Table 2 shows the output plots generated by each method, contributing with different representations of the genes that were differentially expressed. Some of these plots were already used in the “(1) Data Analysis” section (PCA and MDS plots). If the user indicated that samples for a given condition belonged to different batches, the batch error effect correction for several methods will be applied.

**Table 2:** Plots generated by each differential expression package.

Plot/Method	edgeR	limma	NOISeq	DESeq2
Expression	X	X	Yes	X
MA	X	X	X	Yes
MD	X	Yes	Yes	X
Smear	Yes	X	X	X
Volcano	Yes	X	X	X

#### Expression, MA, MD and Smear Plots

These plots depict all expressed genes but those with differential expression are represented in other color than black. Basically, in all of them we can see the distribution of the gene expression according to a certain value. For example, in the expression plot (Supplementary Figure S1) the average expression values for each gene of the compared conditions are plotted and those highlighted in red are genes with a significant difference compared to the rest. In simple terms, the differentially expressed genes are those with outlier mean values.

In the MA-plot (Supplementary Figure S2), the log2 fold change (logFC) expression and the normalized mean counts of each gene in the compared conditions are plotted. Features declared as differentially expressed are highlighted in different colors according to the logFC threshold defined by the user and the expression directionality (UP or DOWN).

The mean-difference (MD) plot (Supplementary Figure S3) shows the average expression (mean: x-axis in limma or D for NOISeq) against logFC (difference: *y*-axis in limma or M for NOISeq). Again, values declared as differentially expressed are highlighted in red.

The smear plot allows to visualize the results of the analysis in a similar manner to the MA-plot, this plot shows the logFC against log-CPM, where genes declared as differentially expressed highlighted in red.

In summary, all these plots compare the expression rate or difference between conditions and the normalized values. The proportion of black and highlighted dots gives an idea of the expression change magnitude between the treatment and the control or untreated conditions.

#### Volcano Plot

**Figure 7:**
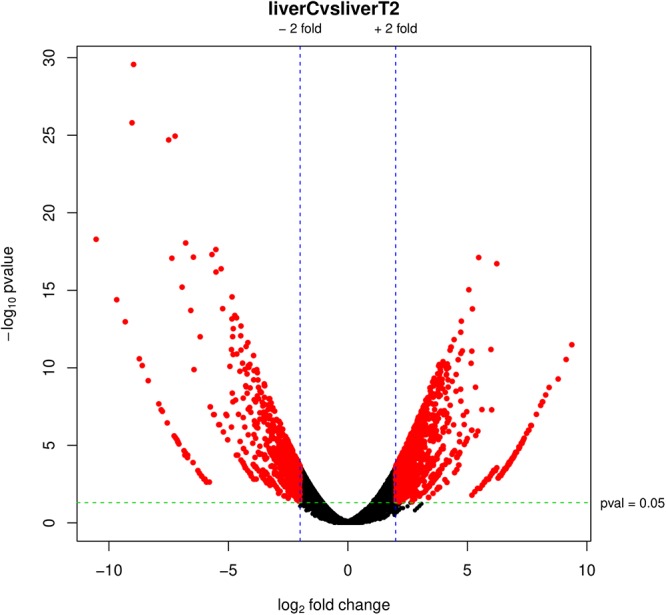
Volcano plot. Red dots represent differentially expressed genes according to the *p*-adj and logFC cut-off values.

Arguably, the volcano plot (Figure 7) is the most popular and probably, the most informative graph since it summarizes both the expression rate (logFC) and the statistical significance (*p*-value). It is a scatter-plot of the negative log10-transformed *p*-values from the gene-specific test (on the *y*-axis) against the logFC (on the *x*-axis). The graph depicts datapoints with low *p*-values (highly significant) appearing toward the top of the plot. The logFC values are used to determine the change direction (up and down) appearing equidistant from the center. Features declared as differentially expressed are highlighted in red, according to the selected cut-off values.

#### Results Integration

**Figure 8:**
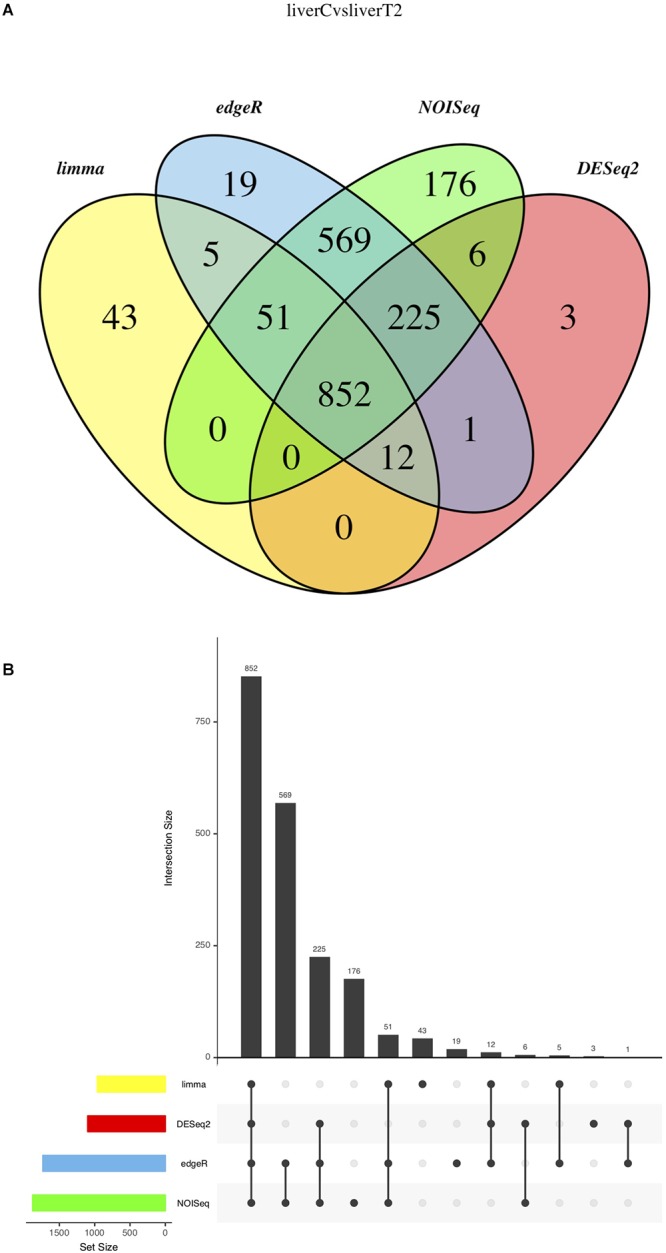
Result Integration summary. **(A)** Venn diagram representing the result intersection for each selected method. **(B)** Upset plot representing the contribution of each selected method.

Finally, the “(3) Results Integration” section of the Analysis Results in the IDEAMEX web page contains several text files and graphs that integrates the results from all selected methods. In Figure 8, we present the results from the C vs. T2 comparison, using a Venn diagram (Figure 8A), upset bar (Figure 8B) and correlograms (Supplementary Figure S5) plots. For the analyzed data, the Venn diagram showed all method intersections and it is observed that 852 genes were validated as differentially expressed by all four methods, being NOIseq the main contributor as also observed in the upset bar plot. It is interesting that limma-Voom reported that 43 genes that no other method found as differentially expressed but agreed with the other methods in 920 genes (5 + 51 + 852 + 12). This gives the option to the user to work with either only the intersection or the union of all methods. However, working with all methods can be overwhelming for the user although using an enrichment analysis using the GO term or metabolic annotation from KEGG could help.

As mentioned, other generated plots are heatmaps (Supplementary Figure S4) and correlogram (Supplementary Figure S5) plots. Since each method has different normalization methods, fold change or statistical metrics (*p*-adj, FDR or Probability) to determine if a gene is differentially expressed, the correlograms can help the user to evaluate the correlation of these values among the different used methods. Also, heatmaps are created to observe samples clustered by their fold change, allowing the user to spot groups of genes with a similar expression change.

Among all the text file results that are explained in detail in the User Manual (Supplementary Material S2), the IntersectTopRegulation.txt file provides the list of all differentially expressed genes with a 0| 1 matrix that can be used select genes depending on how many and which methods reported them as differentially expressed. In the last column of the file, a description of the gene regulation can be found, where is indicated how and in which condition the genes was expressed. Table 3 has a snipped of the liverCvsliverT2_IntersectTopRegulation.txt file where the Regulation structure results is as follows: UP_conditionX_DOWN_conditionY or DOWN_conditionX_UP_conditionY. Therefore, the user can select which genes were up or down regulated in a certain condition and be sure of the directionality of the expression without checking the fold change directionality.

**Table 3:** Example of intersect results table.

	edgeR	limma	NOISeq	DESeq2	Regulation
ENSONIT00000000023	1	1	1	1	DOWN_liverC_UP_liverT2
ENSONIT00000000075	1	1	1	1	DOWN_liverC_UP_liverT2
ENSONIT00000000081	1	1	1	1	DOWN_liverC_UP_liverT2
ENSONIT00000000102	1	1	1	1	UP_liverC_DOWN_liverT2
ENSONIT00000000120	1	1	1	1	DOWN_liverC_UP_liverT2
ENSONIT00000000129	1	1	1	1	DOWN_liverC_UP_liverT2
ENSONIT00000000206	1	1	1	1	DOWN_liverC_UP_liverT2

*Snippet of the liver CvsT2 treatment. Original table is in simple text format. The Regulation column indicates the directionality of the gene expression*.

## Discussion

The IDEAMEX web server is a useful resource for transcriptome experiments designed for differential expression analysis involving several condition comparisons. The methods for differential expression analysis in the workflow, were selected based on their performance in several benchmark analyses since the emergence of RNA-Seq data as a powerful alternative to microarrays (Anders et al., 2013; Soneson and Delorenzi, 2013; Seyednasrollah et al., 2015; Costa-Silva et al., 2017). In particular, our web server uses R packages that use different algorithms and normalization methods giving a broader view of the results with a higher confidence based on their agreement, based on the idea that no statistical modeling can fully capture biological phenomena. In the case of limma and NOIseq, they use non-parametric methods that are statistical techniques for which we do not have to make any assumption of the gene expression; whereas DESeq2 and edgeR use parametric methods assuming a binomial distribution for the data and that no genes are differentially expressed.

Once the user had loaded the input data in the right format, our server allows the user to design which comparisons will be made and which cut-off values will be used, instead of running an all-vs.-all comparison and default parameters for each package. For parametric methods like edgeR and DESeq2, the FDR and *p*-adj values are the statistical parameters that define the probability of a gene to be differentially expressed in a multiple comparison and are used to define if a gene was differentially expressed or not from the statistical point of view. However, other parameters such as the CPM or logFC can have a biological meaning and also can be used a cut-off value. Is not straightforward how to select which cut-off values will be the best for a certain experiment but IDEAMEX allow users to try many combinations of them by running the comparisons several times and inspecting the different results.

Is very important that the user select which comparisons have a sense in terms of their experimental design. For example, in this work we used three conditions where one was used as a control to study the effect of two thyroid hormones treatments in tilapia liver (T2 and T3). The comparison between T2 and T3 has to be performed by comparing the results from comparing each one to the control or untreated condition. A direct comparison between T2 and T3 could miss several results since even if we can observe a gene with a certain expression change, the difference could not be statistically significant. Let’s say that “gene A” has a differential expression of 10 times in T2 vs. C comparison and of 12 times in the T3 vs. C comparison. Roughly, the difference between T2 vs. T3 comparison for the same gene, will be 2 times which might not be statistically significant. For this reason, is very important to select the which comparisons make sense, instead of performing all possible comparison.

The results in the “Data Analysis” section, are several plots that allow the user to inspect the distribution of their data based on different metrics. This quality control check point is very important, since biological data tend to be very noisy. It is expected that the data from biological replicates within a certain condition, will have the same distribution and a similar trend than those in other conditions. In particular, PCA and MDS plots allow the users to see if biological replicates of a certain condition are grouped together and if each condition forms a separate group. In this particular case, it was known that the samples didn’t present any batch effect but as observed in Figure 6, there is some dispersion between samples. It is not trivial to determine if samples present a dispersion attributable to a batch effect. Therefore, it is important to obtain the information regarding the sample preparation to discriminate between high “biological” variability and “noise” from batch effect.

The distance or dispersion of the replicates and groups indicates how reproducible was the tested condition in different individuals or how variable were individuals despite the treatment. The more replicates available, the better statistical significance is observed. Having very disperse groups or samples from different conditions grouping together, should be considered as noisy or highly variable results that can skew the analysis and lead to misinterpretation of the experiments. However, NOIseq could be a good option when no biological replicates are available and as reported elsewhere, it delivers reliable results that have been confirmed by using quantitative PCR (qPCR) reactions.

The results from different methods are not mutually exclusive. From the statistical point of view, one of them, neither or all may be true. Therefore, working with the intersection or the union of all results is a decision that the user has to evaluate after exploring them based not only on the statistical significance but on the biological meaning that will depend on the gene annotation. The main problem with all statistics is the “fakeness” and misrepresentation of the results. However, if four different methods agreed with a certain result it could be assumed that those genes are differentially expressed, bearing in mind that an experimental orthogonal validation using a different technology like qPCR, should be necessary to confirm the result.

In the “Results Integration” section, there are several text lists and graphs that can guide the users to make sense out of the results from their experiments. As mentioned, the Venn diagram (Figure 8A) shows the intersection and union of the selected different methods. The user can choose one or more methods by evaluating the agreement between them since one method could generate either an overwhelming amount of results or very few of them. In the former case, the user can choose to work with the intersection of all methods or in the latter case, the union will provide the maximum amount of reported results.

In this work, we provide heatmaps and correlograms for different values obtained from each method. For example, heatmaps (Supplementary Figure S4) are useful to spot gene clusters with the same fold change pattern, suggesting that those genes could belong to a certain pathway of are regulated by the same mechanism. However, users have to be very careful when determining gene clusters since there is no straightforward method to do so. Defining the cluster size is not trivial and usually is a trial and error process. In terms of novelty, the most interesting plot could be the statistical parameter correlogram (Supplementary Figure S5), where the threshold values such as p-adj (limma-Voom and DESeq2), FDR (edgeR) and Prob (NOISeq) values are correlated. To our knowledge, this correlation has not been reported in other studies. Surprisingly, methods usually correlate very well since the statistical threshold denotes the error probability of each result. In our experience, we have observed that NOISeq is the method with lower correlation regarding the error probability since this is calculated using a very different approach (Tarazona et al., 2011) compared to the rest of the methods. However, is somehow refreshing that all methods present a good correlation, suggesting that are consistent identifying differentially expressed genes and those with no significant change, despite using different statistics.

Finally, there are several other methods to continue the differential expression analysis, that can help users to put their results in a certain biological context. Probably the most popular methods are those based on Gene Ontology (GO) terms enrichment (Maere et al., 2005; Eden et al., 2009; Reimand et al., 2016) which will require of a well curated gene annotation. Other enrichment methods like Gene Set Enrichment Analysis (GSEA) determine whether a defined set of genes shows statistically significant based on molecular signatures (Subramanian et al., 2007; Liberzon et al., 2011) or metabolic pathway enrichment analysis (Luo et al., 2009; Liu et al., 2017; Ulgen et al., 2018) can provide a better picture of the biological meaning of the observed changes in gene expression for a given treatment or condition. These enrichment methods along with the heatmaps, can help the researcher to spot regulation networks or pathways which could be subject to further studies.

## Conclusion

We consider that the IDEAMEX web server can help other researchers with no previous bioinformatic knowledge, to perform their analyses in a simple manner. Also, more experienced users with some bioinformatics skills can use the results and perform a more detailed analysis and a different integration of them, since all the results are provided in simple text files which are very convenient to parse and handle using regular expression searches.

## Data Availability

The datasets analyzed for this study can be found in the NCBI SRA repository (https://submit.ncbi.nlm.nih.gov/subs/sra/), under the SRA identifiers: SRX2630485, SRX2630486, SRX2630487, SRX2630488, SRX2630489, and SRX2630490.

## Author Contributions

VJ-J and LV-A developed the web deployment and scripts for the IDEAMEX server. VJ-J, LV-A, and AS-F conceived the web server workflow. AS-F wrote the manuscript. All authors read and authorized the publication of this manuscript.

## Conflict of Interest Statement

The authors declare that the research was conducted in the absence of any commercial or financial relationships that could be construed as a potential conflict of interest.

## Supplementary Material

FIGURE S1 Liver C vs. T3 MA plot.

FIGURE S2 Liver C vs. T3 MD plot.

FIGURE S3 Liver C vs. T3 smear plot.

FIGURE S4 Liver C vs. T3 heatmap.

FIGURE S5 Liver C vs. T3 statistic parameter correlogram.

MATERIAL S1 Raw count table.

MATERIAL S2 IDEAMEX user manual.
