# Supplementary figures and images for: Integrative Differential Expression Analysis for Multiple EXperiments (IDEAMEX): A Web Server Tool for Integrated RNA-Seq Data Analysis
Source: Front Genet. 2019 Mar 29;10:279. doi: 10.3389/fgene.2019.00279 (PMC6450261; doi:10.3389/fgene.2019.00279)

# liverCvsliverT3

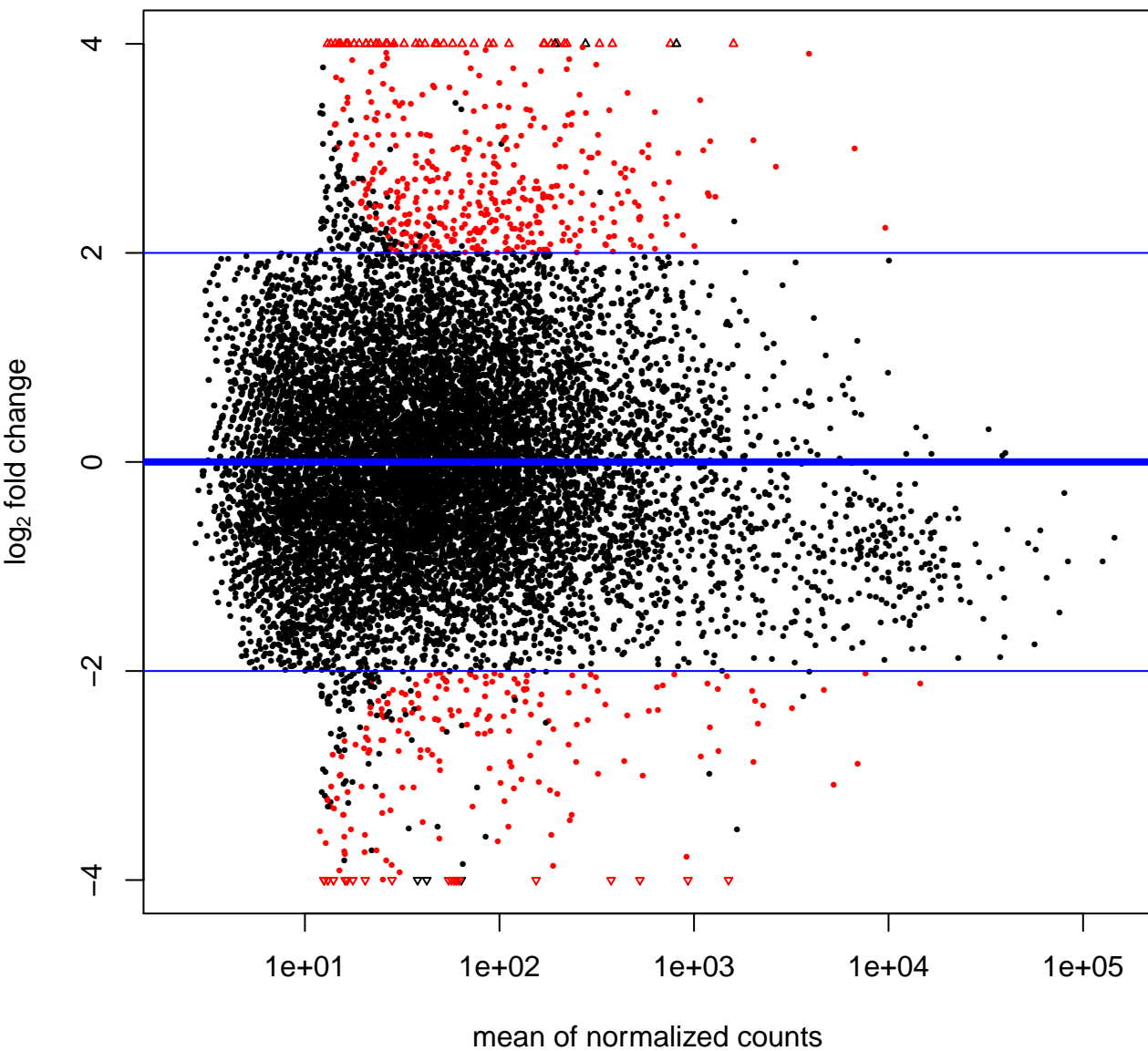

Supplement: FIGURE S1 — Liver C vs. T3 MA plot. [file Image_1.pdf]

# liverCvsliverT3

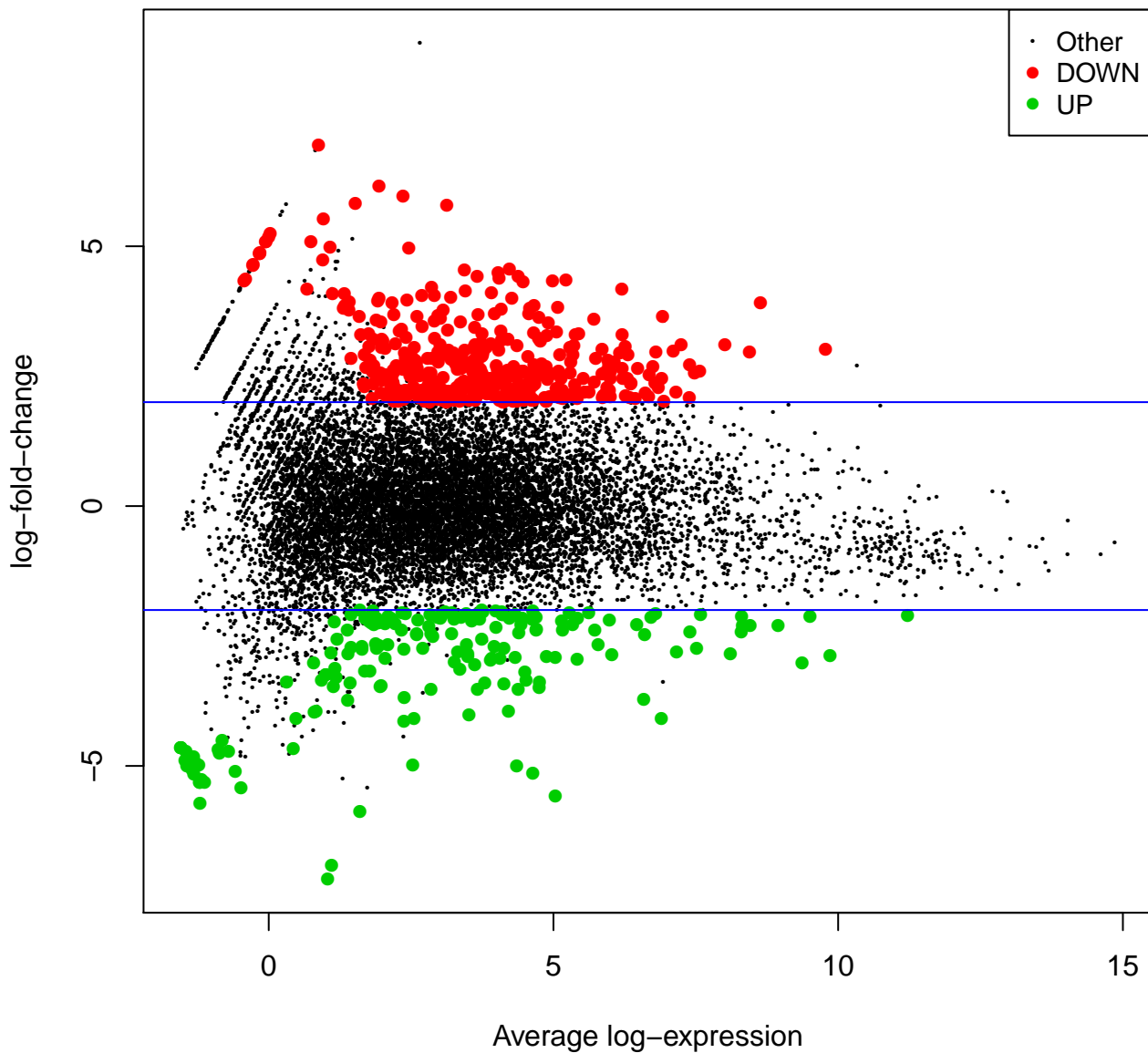

Supplement: FIGURE S2 — Liver C vs. T3 MD plot. [file Image_2.pdf]

# liverCvsliverT3

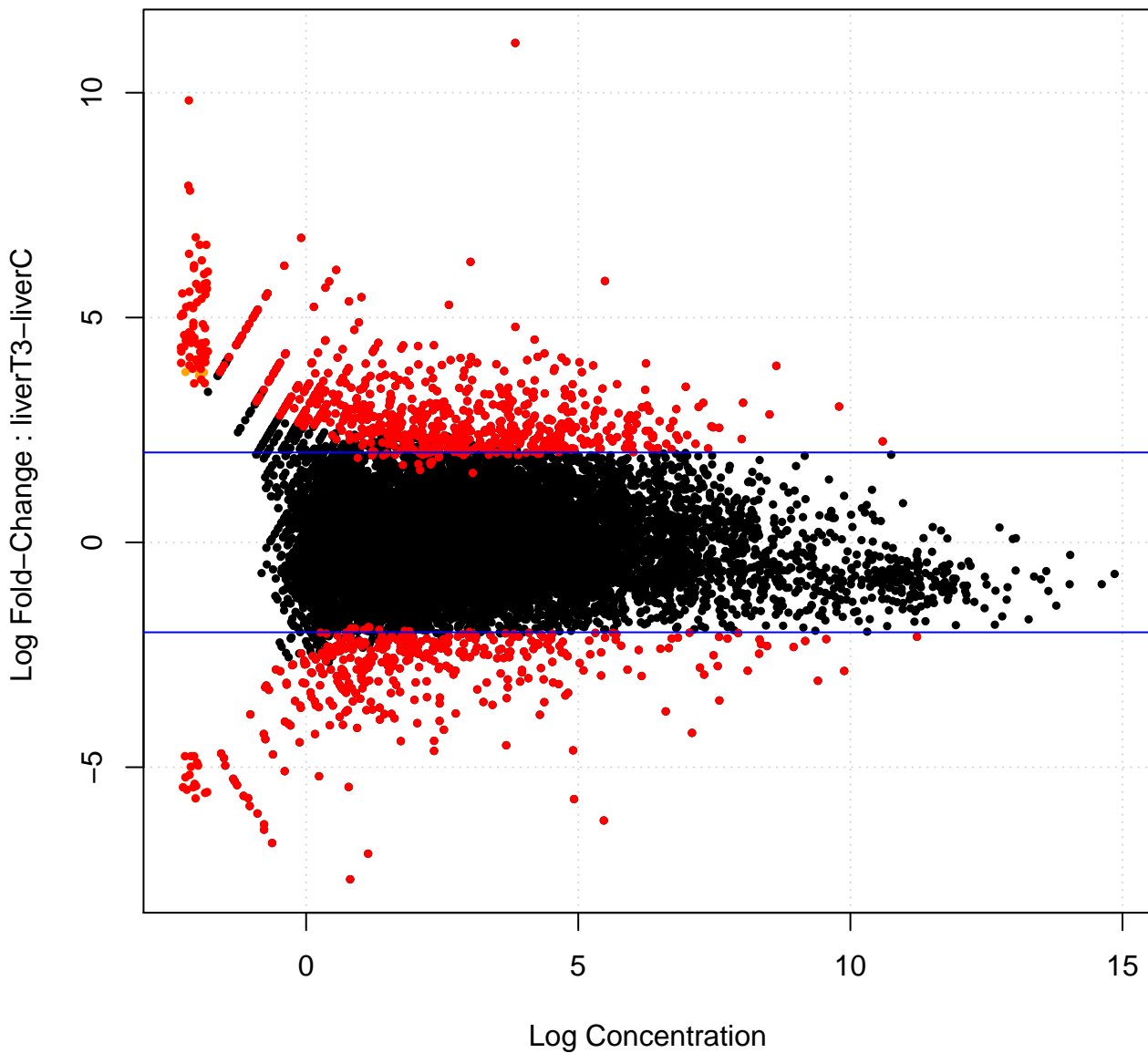

Supplement: FIGURE S3 — Liver C vs. T3 smear plot. [file Image_3.pdf]

# liverCvsliverT2

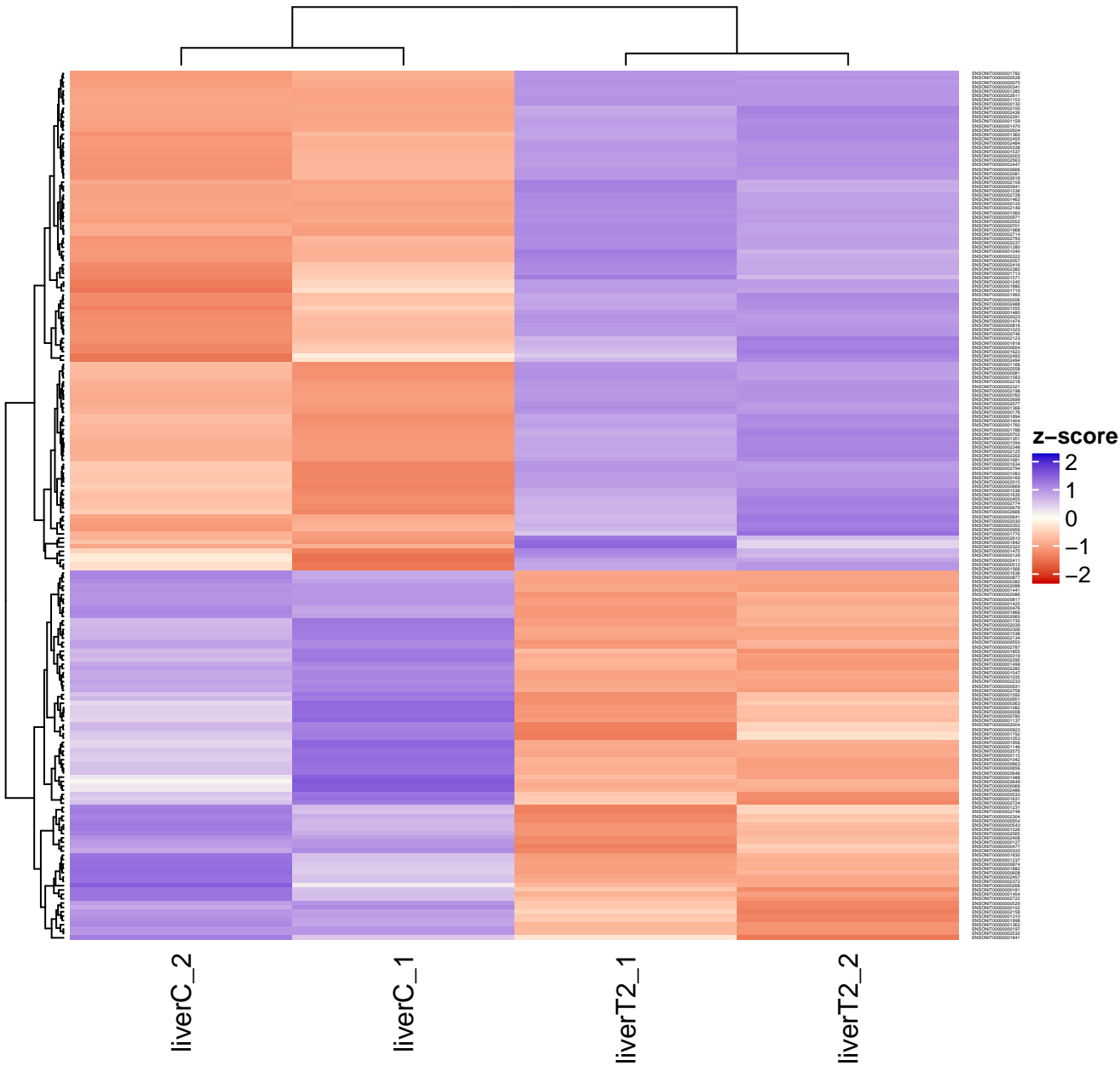

Supplement: FIGURE S4 — Liver C vs. T3 heatmap. [file Image_4.pdf]

# Pval correlation liverCvsliverT2

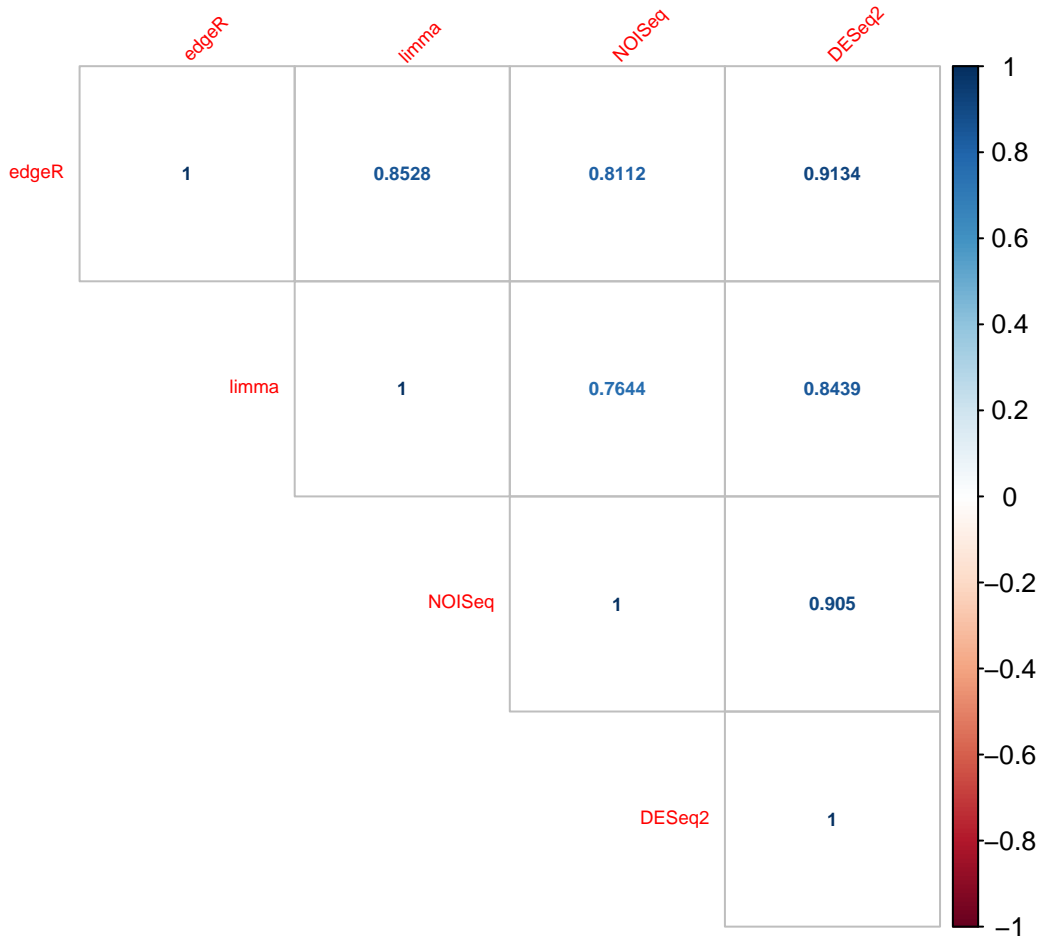

Supplement: FIGURE S5 — Liver C vs. T3 statistic parameter correlogram. [file Image_5.pdf]
