# Supplementary material for: Integrative Differential Expression Analysis for Multiple EXperiments (IDEAMEX): A Web Server Tool for Integrated RNA-Seq Data Analysis
Source: Front Genet. 2019 Mar 29;10:279. doi: 10.3389/fgene.2019.00279 (PMC6450261; doi:10.3389/fgene.2019.00279)
Supplement: MATERIAL S2 — IDEAMEX user manual. [file Data_Sheet_2.PDF]

# IDEAMEX: Integrative Differential Expression Analysis for Multiple EXperiments

User's Guide

*Verónica Jiménez-Jacinto*
*Alejandro Sánchez-Flores*
*Leticia Vega-Alvarado*

*Last revised February 2019*

## Contents

**1 Introduction** **2**
1.1 Citation . . . 2

**2 Accessibility** **2**

**3 Starting an analysis** **3**
3.1 Raw count table format . . . 3
3.1.1 Sample names format . . . 4
3.2 Uploading data file . . . 4
3.3 Methods selection . . . 4
3.4 Samples names validation and edition . . . 5
3.5 Comparison definition . . . 6
3.5.1 Setting cutoffs parameters . . . 6
3.6 Submit the analysis . . . 6

**4 Data Analysis** **7**
4.1 Pseudo-counts . . . 7
4.2 Plots . . . 8
4.2.1 Boxplot . . . 8
4.2.2 Density plots . . . 8
4.2.3 Count Per Million plot . . . 8
4.2.4 Principal Components Analysis plot . . . 8
4.2.5 Multi-Dimensional Scaling plot . . . 10

**5 Differential Expression Analysis** **10**
5.1 Raw data filtering . . . 11
5.1.1 Count Per Million . . . 11
5.2 Evaluation of the differential expression . . . 11
5.3 Output Plots . . . 11
5.3.1 Expression plot . . . 11
5.3.2 MA plot . . . 12
5.3.3 MD plot . . . 12
5.3.4 MDS plot . . . 12
5.3.5 PCA plot . . . 14
5.3.6 Smear plot . . . 14
5.3.7 Volcano plot . . . 14
5.4 Output text files . . . 14

**6 Result integration** **16**
6.1 Integration output plots . . . 18
6.1.1 Venn diagram . . . 18
6.1.2 Upset plot . . . 18
6.1.3 Correlograms . . . 18
6.1.3.1 pvalues correlogram . . . 18
6.1.3.2 logFC correlogram . . . 18
6.1.3.3 Abundance correlograms . . . 18
6.1.4 Heatmaps . . . 20
6.2 Integration output text files . . . 20

**7 Case of studies** **20**
7.1 Comparison between two groups . . . 20
7.2 RNA-Seq with batch effect . . . 25

**References** **30**

# 1 Introduction

IDEAMEX (Integrative Differential Expression Analysis for Multiple EXperiments) is a web server where users can run simultaneously, the best Bioconductor (Huber W 2015) packages for RNA-seq differential expression analysis. The web server also integrates the results from each package, so users can select between the intersection or union of all results. The sole input for the IDEAMEX pipeline, is a raw count table in simple text format, for as many desired replicates and conditions, allowing the user to select which conditions will be compared, according to the biological design behind the experiment. The process consists of three main steps 1) Data Analysis: that allows a preliminary analysis for data quality control based on the data distribution per sample, using different types of graphs; 2) Differential expression: performs the differential expression analysis using the bioconductor packages: edgeR (Robinson MD 2010), limma - voom (Ritchie ME 2015), DESeq2 (Love MI 2014) and NOISeq (D. J. Tarazona S Garcia-Alcalde F 2011), and generates reports for each method; 3) Result integration: the integrated results are reported using Venn diagrams, heatmaps, correlograms and text lists where differentially expressed genes are reported, according to the cutoff lines defined by the user. Our server allows an easy and friendly visualization of results, providing an easy interaction during the analysis process, as well as error tracking and debugging by providing output log files.

## 1.1 Citation

IDEAMEX: Integrated Diferencial Expresion Analysis for Multiple EXperiments (http://www.uusmb.unam.mx/ideamex), Jimenez-Jacinto Verónica, Sanchez-Flores Alejandro, Vega-Alvarado Leticia.

For citation when you publish results obtained using the IDEAMEX Website.

Since IDEAMEX provides interfaces to differential expression analysis methods implemented in other R packages, take care to cite the appropriate references (see the reference manual for more information).

IDEAMEX is free for use by academic users. If you want to use it for commercial settings, please contact Leticia Vega Alvarado at: leticia.vega@icat.unam.mx.

# 2 Accessibility

IDEAMEX can be accessed directly through the link:

http://www.uusmb.unam.mx/ideamex

Once the user accesses the WEB site, will see the IDEAMEX home page (Figure 1). Home page contains the following fields and buttons:

1. email. Valid email for confirmation of your input data.
2. Select file. Browse and select from your computer a raw count table file
3. Submit. Starting analysis
4. Examples of data sets. RNA-Seq data with and without batch effects.
5. User's Guide
6. Exit

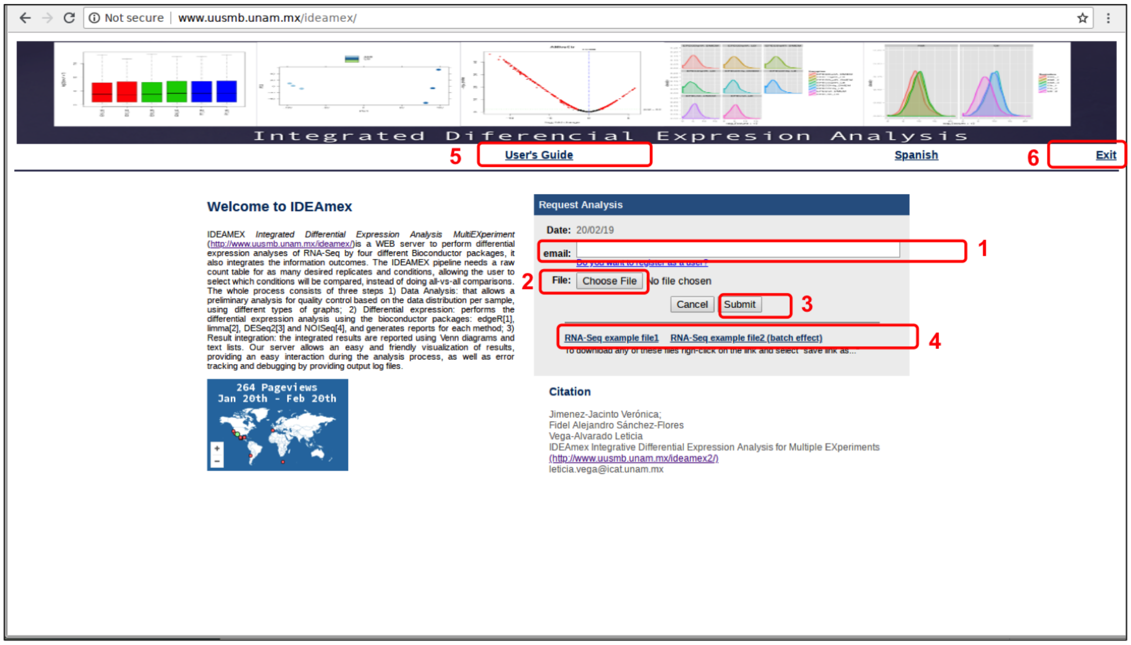


Figure 1: IDEAMEX home page

# 3 Starting an analysis

IDEAMEX web site requires two types of data to perform the analysis. The first is a valid email address ( that allows us to create you a workspace where you can store your analysis data on our site) and the second one is a file with the raw count table in text format.

## 3.1 Raw count table format

The raw count table file should be in tabular text format with the row names to list the regions of interest (gen IDs, trascripts among others) and column names to list the sample IDs. In the IDEAMEX principal web page, we provide two examples of datasets (RNA-Seq file 1 and RNA-Seq file 2). By clicking in any of the example files, you will see the data information of the example. Please, save each of the examples to a local file (using Ctrl+S) or righ-click on the link and select "save link as..." in your computer to use them as input.

The RNA-Seq data of the example file 1, was taken from the Pasilla Bioconductor library produced by Brooks et al (Brooks AN 2010), taking in account only the gene level counts. This dataset contains RNA-seq count

data for treated and untreated cells from the S2-DRSC cell line. Some rows of the file are shown in Table 1. The section 7.1: Comparison between two groups shows this example, fully worked in IDEAMEX.

Table 1: Raw count table example file

| | untreated_1 | untreated_2 | untreated_3 | untreated_4 | treated_1 | treated_2 | treated_3 |
|---|---|---|---|---|---|---|---|
| **FBgn0000003** | 0 | 0 | 0 | 0 | 0 | 0 | 1 |
| **FBgn0000008** | 92 | 161 | 76 | 70 | 140 | 88 | 70 |
| **FBgn0000014** | 5 | 1 | 0 | 0 | 4 | 0 | 0 |
| **FBgn0000015** | 0 | 2 | 1 | 2 | 1 | 0 | 0 |
| **FBgn0000017** | 4664 | 8714 | 3564 | 3150 | 6205 | 3072 | 3334 |
| **FBgn0000018** | 583 | 761 | 245 | 310 | 722 | 299 | 308 |

The RNA-Seq data of the example file 2, was taken from the NBPSeq CRAN package (Di Y 2014). This dataset cointains the Arabidopsis thaliana RNA-Seq data described by Cumbie et al. (Cumbie JS 2011), comparing ΔhrcC challenged and mock-inoculated samples (Cumbie JS 2011). In this case, the samples were collected in three batches. The section 7.3: RNA-Seq with batch effect shows the example file 2, fully worked in IDEAMEX.

### 3.1.1 Sample names format

The conditions of the experiments are obtained automatically from the samples names in the raw count table and for this reason they must have the following format:

nameCond1_1, nameCond1_2, . . . , nameCond1_n,

nameCond2_1, nameCond2_2, . . . , nameCond2_m,

. . .

nameCondk_1, nameCondk_2, . . . , nameCondk_p

In the example file 1 the samples names are:

untreated_1 untreated_2 untreated_3 untreated_4

treated_1 treated_2 treated_3

Therefore the condition of the experiments, in example file 1, will be: **untreated** and **treated**.

## 3.2 Uploading data file

The user will use the "Choose file" button on the main page to select the file with the count table text file. Once the file was uploaded and the email was provided, it is necessary to click on the submit button, to continue with the analysis methods selection.

## 3.3 Methods selection

After the raw count table has been read, the application will show a window for the Methods selection (Figure 2). One or several methods can be checked at the same time. It is important to mention that at least one differential expression method must be selected, otherwise an error message will appear. It is possible to select all the methods by checking the "All" box or deselect the entire selection by checking the "Clear" box. When the methods have already been selected, we click on the continue button.

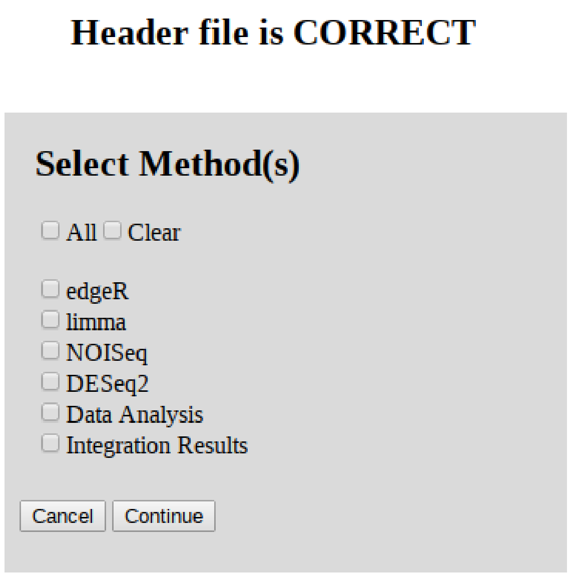


Figure 2: Methods selection

### 3.4 Samples names validation and edition

Following Methods selection, the application will display a table with the names of the samples(Figure 3), which can be edited if required (see Sample names format).

In addition, if you are interested in performing the differential expression analysis considering a possible batch effect (Goh WWW 2017) in your samples, you can use the **"Batch"** column to assign each sample to a lot, as shown in Figure 3(b). Otherwise leave this column empty, as shown in figure 3(a).

Click on the continue button to define the comparisons between conditions.

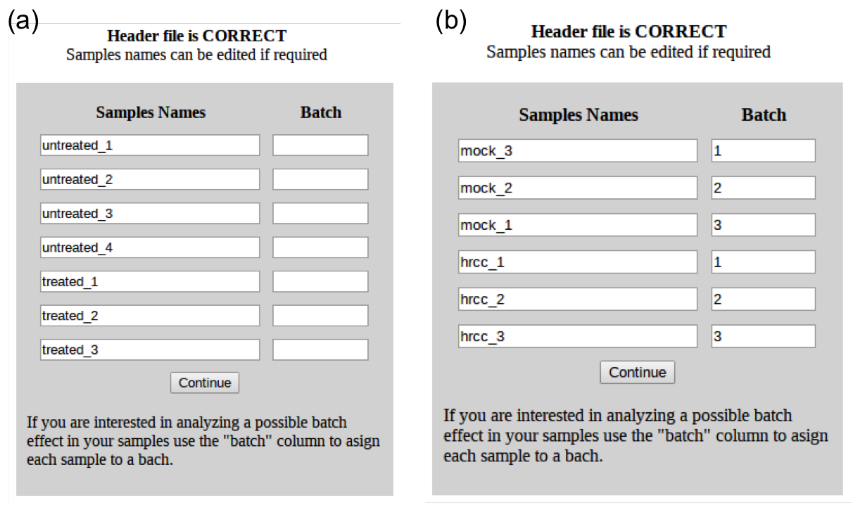


Figure 3: Samples name validation. (a) samples without batch effects, (b) samples with batch effects

## 3.5 Comparison definition

Once the DE methods have been selected (see Methods selection), the user can select the specific comparisons of interest from the permutations of all pairwise comparisons, as shown in Figure 4. The section 7.2 gives a fully worked example of [Pairwise comparisons between more than two groups].

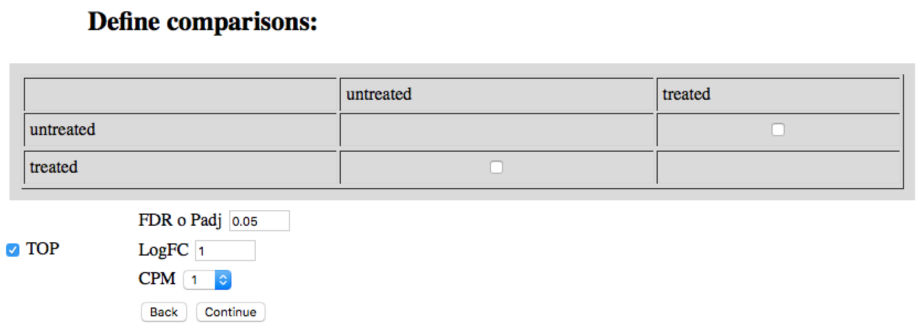


Figure 4: Comparison Selection

### 3.5.1 Setting cutoffs parameters

Also in this step some parameters like FDR(False Discovery Rate) or padj(PValue Adjusted), logFC (log fold change) and CPM (Count Per Million) cutoff values can be specified in order to filter the DE final results. The default values are 0.5 for the FDR/padj, 1 for the logFC and 1 for CPM. Please, adjust these values according to your experimental needs.

FDR/padj and logFC values will be used to obtain the differentially expressed genes for each of the methods. That is, the differentially expressed genes will be those with values greater than or equal to the selected logFC and lower than or equal to the FDR/pdajust selected value.

If the user checks the TOP box, the TOP files containing the information of differentially expressed genes are generated by each method (see Output text files section). In addition, these information is used for the Result integration module.

Moreover, the CPM cut-off value is used to filter the genes with very low counts across all libraries. (see section Raw data filtering).

Finally click submit to start the analysis.

## 3.6 Submit the analysis

Once the analysis process has been requested, the execution of the analysis starts and the link where the results will be available will be displayed, as shown below:

> The results of the analysis requested can be consulted on:
>
> http://zazil.ibt.unam.mx/ideamex/example125850/index.php

You will also receive a notification email with the link. Click on the link in the message to access your results. It is important to mention that this link is the only way of accessing the results. On the other hand, if the user tries to consult the analysis outcomes and these are not available yet, the application will display a message as shown in the Figure 5.

Once the analysis is finished, three outcomes are generated: Data Analysis, Differential Expression Analysis and Result integration, see Figure 6. In the following sections each type of results is explained

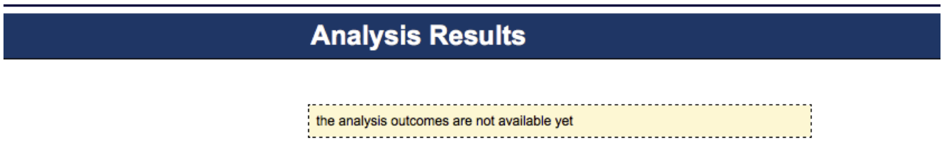


Figure 5: Warnning message

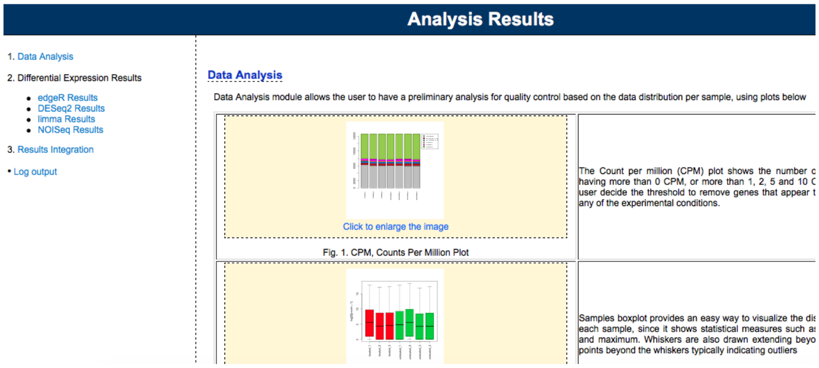


Figure 6: Results Outcomes

# 4 Data Analysis

Data quality assessment and exploration are essential steps of any data analysis. In gene expression analysis, only a small fraction of genes are expected to show Differential Expression (DE) between experimental conditions. However, a global overview of all the analyzed genes should provide, if it exists, some evidence of the evaluated experimental conditions. For example, sample replicates (technical or biological) for a given condition, should have similar distribution but different to replicates from another condition. In this evaluation, is possible to detect problems such as batch effects or probable sample mislabeling (Gonzalez 2014).

The Data Analysis module allows the user to have a preliminary visual analysis for quality control based on the data distribution per sample, using different types plots (Boxplot, Density plots, Principal Components Analysis plot (PCA), Multi-Dimensional Scaling plot (MDS)).

## 4.1 Pseudo-counts

Data exploration and visualization is worked with transformed versions of the count data, i.e. with pseudo-counts. As the count values distribution is highly skewed, then a log2 transformation is applied to roughly help normalize the distributions. The pseudo-counts are computed just to make the graphs.

It is important to mention that the boxplot, PCA and MDS graphs are ploted with the pseudo-counts and are also plotted with the normalizaed data using the Trimmed Mean of M-values (TMM) method (Brooks AN 2010).

## 4.2 Plots

### 4.2.1 Boxplot

Samples boxplot provides an easy way to visualize the distribution of Pseudo-counts in each sample, since it shows statistical measures such as median, quartiles, minimum and maximum. Whiskers are also drawn extending beyond each end of the box with points beyond the whiskers typically indicating outliers. Figure 7, shows the boxplot related to the example dataset.

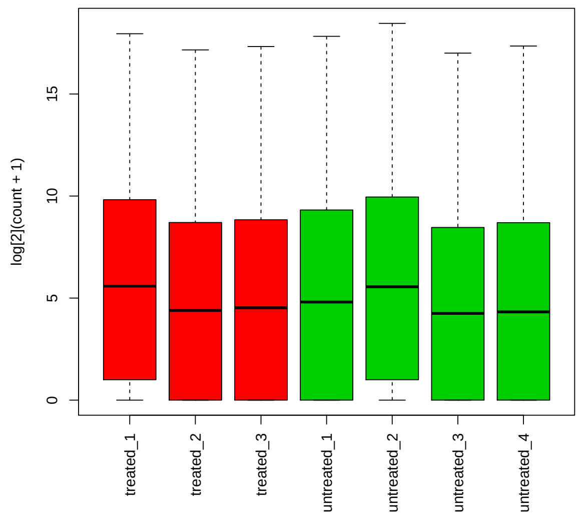


Figure 7: Boxplot example with pseudocounts

### 4.2.2 Density plots

Pseudo-counts distributions can also be summarized by means of a density plot. Density plot provide more detail by enabling, for example, the detection of a dissimilarity in replicates behave. Figure 8, shows a density plot example.

### 4.2.3 Count Per Million plot

The Count Per Million (CPM) (Robinson MD 2010) plot shows the number of genes within each sample having more than 0 CPM (Count Per Million), or more than 1, 2, 5 and 10 CPM. This plot could help the user decide the threshold to remove genes that appear to be very lowly expressed in any of the experimental conditions. (T. D. Tarazona S Furio-Tari P 2015). Figure 9.

### 4.2.4 Principal Components Analysis plot

This type of plot is useful for visualizing the overall effect of experimental covariates and batch effects (Love MI 2014). In the context of RNA-Seq analysis, PCA essentially clusters samples by groups of the most significantly deregulated genes. Clustering first by the most significant group, then by progressively less significant groups. Figure 10.

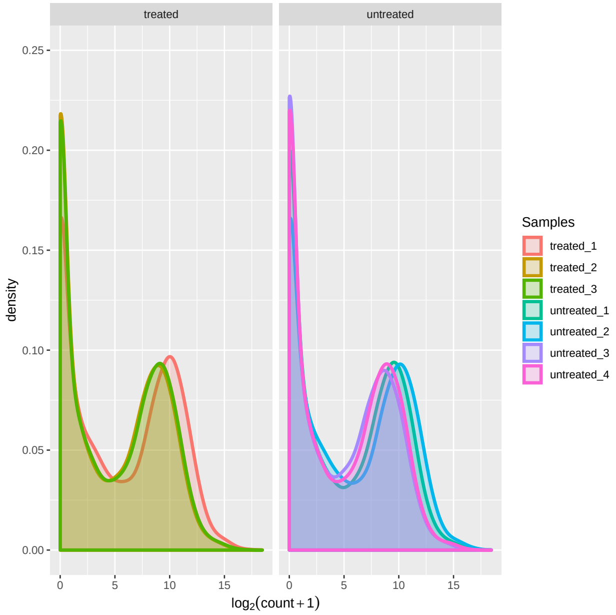


Figure 8: Density plot example with pseudocounts

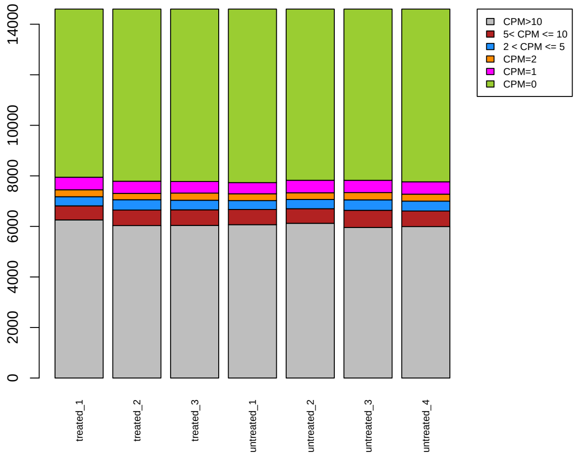


Figure 9: Count Per Million plot

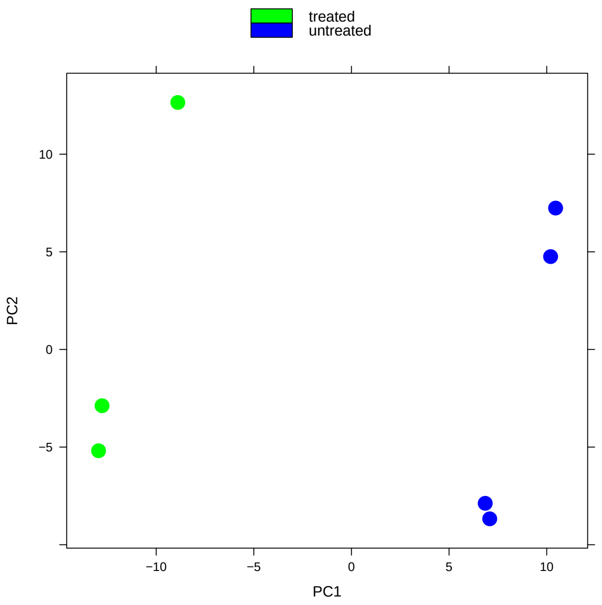


Figure 10: Principal components (PCA) graphic

#### 4.2.5 Multi-Dimensional Scaling plot

Multidimensional Scaling (MDS) is a technique that is used to create a visual representation of the pattern of proximities (similarities, dissimilarities, or distances) among a set of objects(Gonzalez 2014). In the context of RNA-Seq analysis, MDS plot shows variation among RNA-seq samples, distance between sample labels indicates dissimilarity. If the experiment is well controlled and has worked well, what we hope to see is that the greatest sources of variation in the data are the treatments/groups we are interested in (Robinson MD 2010). Figure 11.

## 5 Differential Expression Analysis

The "Differential Expression Analysis" (DEA) could be performed using one or many of the following the bioconductor packages: edgeR, limma-vomm, DESeq2 and NOISeq. Table 2 shows the principal feature used by each method. The DEA module is divided in two steps to perform the analisys. The first step is the Raw data filtering and the second step is the evaluation of the differential expression using the different selected methods.

Table 2: Principal features of methods

| **Package** | Version | Normalize | Comparison |
|---|---|---|---|
| **edgeR** | 3.24.1 | TMM | Replicates/without Replicates |
| **limma-vomm** | 3.38.3 | log2-counts per million | Replicates |
| **DESeq2** | 1.22.1 | DESeq2-default method | Replicates/without Replicates |
| **NOISeq** | 2.26.0 | TMM | Replicates/without Replicates |

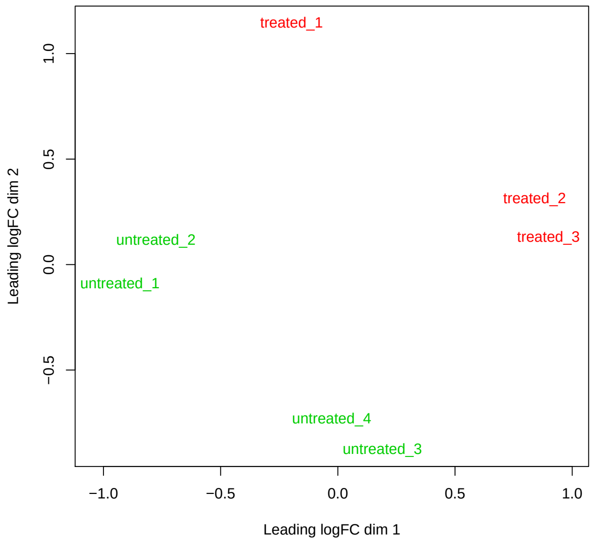


Figure 11: Multidimensional Scaling (MDS) graphic

## 5.1 Raw data filtering

Since genes with very low counts across all libraries provide little evidence for differential expression and they could interfere with some of the statistical approximations, it is advisable to filter them.

In this step raw data are filter based on the CPM (Count Per Million) function from the edgeR library. Genes without at least a CPM value cutoff in n of the samples, where n is the size of the smallest group of replicates, are removed from the analysis (Anders S 2013).

### 5.1.1 Count Per Million

The Counts Per Million (CPM) (Robinson MD 2010) is calculated as the raw counts divided by the library sizes and multiplied by one million.

## 5.2 Evaluation of the differential expression

When the differential expression analysis is performed, graphic and text files are generated with the results of the analysis for each selected method. The information on the results files is detailed below.

## 5.3 Output Plots

The description of the output plots that are generated during the evaluation of the differential expression is shown in Table 3.

### 5.3.1 Expression plot

In this graph the average expression values of each condition are plotted and the features declared as differentially expressed are highlighted in red (D. J. Tarazona S Garcia-Alcalde F 2011). (see Figure 12).

Table 3: Plots

| **Plot** | edgeR | limma | NOISeq | DESeq2 |
|---|---|---|---|---|
| **Expr** | X | X | Yes | X |
| **MA** | X | X | X | Yes |
| **MD** | X | Yes | Yes | X |
| **MDS** | Yes | Yes | X | X |
| **PCA** | X | X | Yes | Yes |
| **Smear** | Yes | X | X | X |
| **Volcano** | Yes | X | X | X |

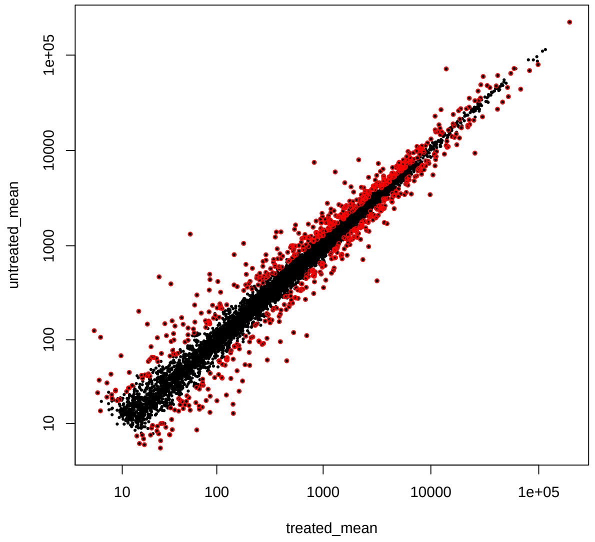


Figure 12: Expression plot

#### 5.3.2 MA plot

This plot represents each gene with a dot. The x axis is the average expression over the mean of normalized counts, the y axis is the log2 fold change between conditions. Features declared as differentially expressed are highlighted in red (Gonzalez 2014). (see Figure 13)

#### 5.3.3 MD plot

The mean-difference (MD) plots show average expression (mean: x-axis in limma or D for NOISeq) against log-fold-changes (difference: y-axis in limma or M for NOISeq). Features declared as differentially expressed are highlighted. (see Figure 14).

#### 5.3.4 MDS plot

In the context of RNA-Seq analysis, MDS plot shows variation among RNA-seq samples, distance between sample labels indicates dissimilarity. If the experiment is well controlled and has worked well, what we hope to see is that the greatest sources of variation in the data are the treatments/groups we are interested in (Gonzalez 2014). (see Figure 15).

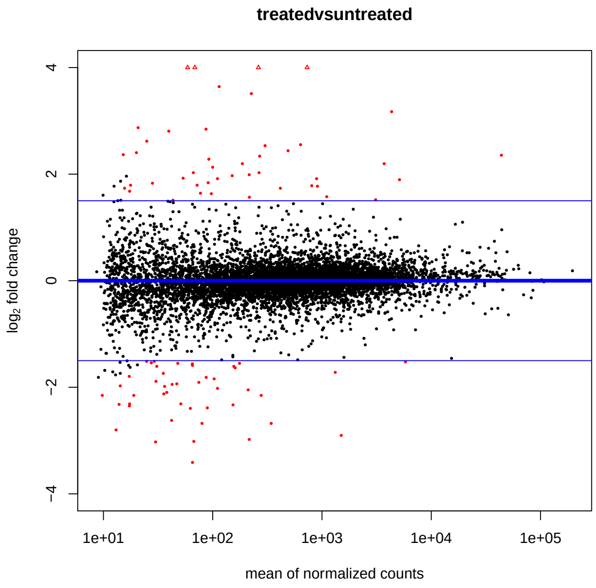


Figure 13: MA plot

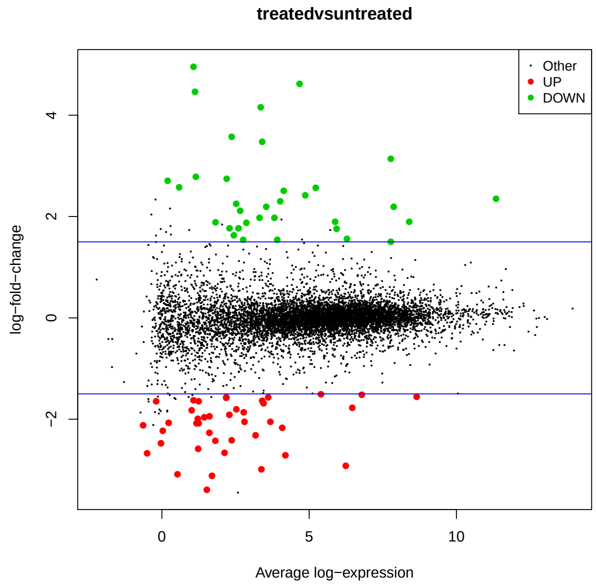


Figure 14: MD plot

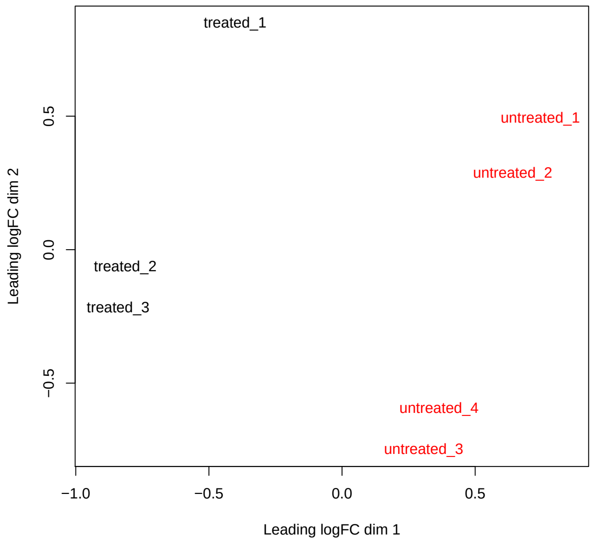


Figure 15: MDS plot

#### 5.3.5 PCA plot

This type of plot is useful for visualizing the overall effect of experimental covariates and batch effects (Love MI 2014). (see Figure 16).

#### 5.3.6 Smear plot

The Smear plot allows to visualise the results of a DE analysis. In a similar manner to the MA plot, this plot shows the log-fold change against log-counts per million, with features declared as differentially expressed highlighted in red. (see Figure 17).

#### 5.3.7 Volcano plot

The Volcanoplot is a graph that summarizes both fold-change and the p-value. It is a scatter-plot of the negative log10-transformed p-values from the gene-specific test (on the y-axis) against the log2 fold change (on the x-axis). This results in datapoints with low p-values (highly significant) appearing towards the top of the plot. The log2 of the fold-change is used so that changes in both directions (up and down) appear equidistant from the center (Gonzalez 2014). Features declared as differentially expressed are highlighted in red. (see Figure 18).

### 5.4 Output text files

For each comparison between groups severals text files are generated, the information of the files are described in table 4. The specific information of all methods is shown in Table 5.

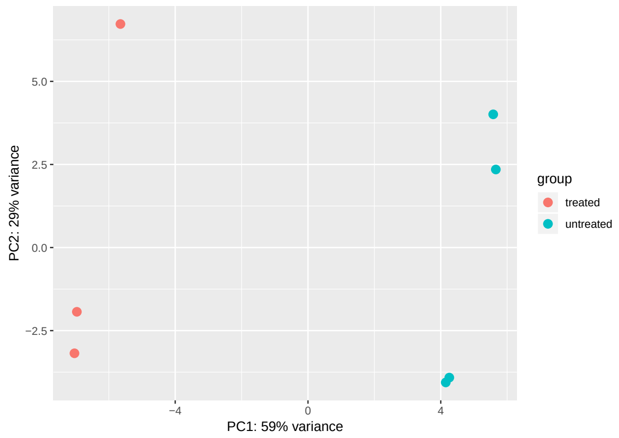


Figure 16: PCA plot

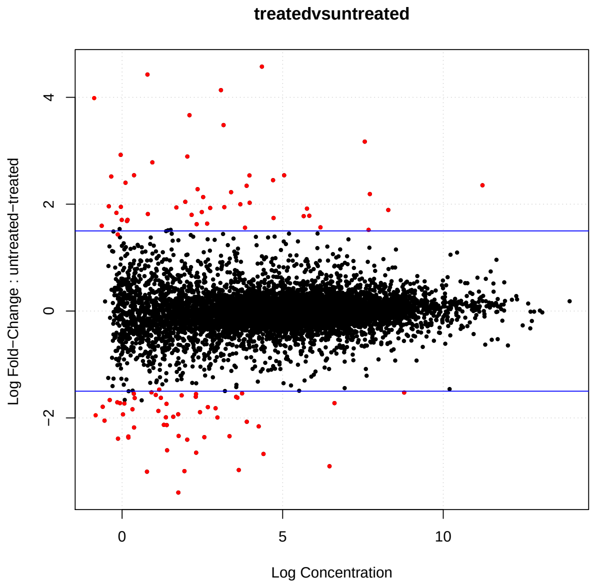


Figure 17: Smear plot

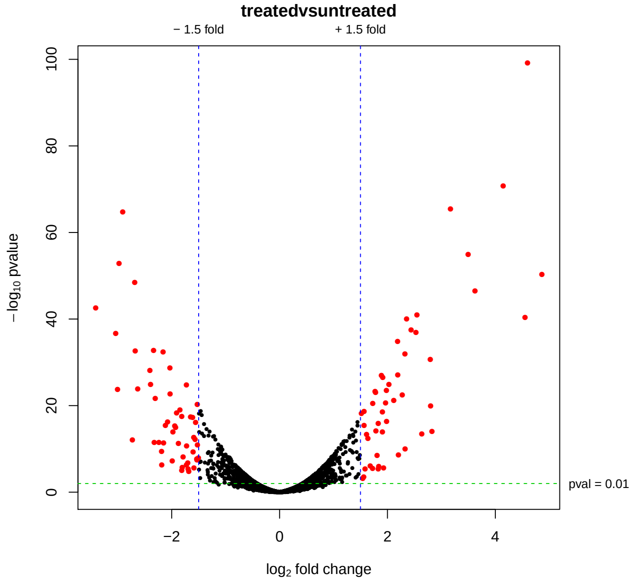


Figure 18: Volcano plot

Table 4: Output files table

| **Files** | Description |
|---|---|
| **AvsB.txt** | Table containing the DE results of all genes, including fold change estimates and p-values. Table 5 shows the specific information of the file. |
| **AvB__TOP.txt** | Table containing DE results only of the differentially expressed genes (considering the established cutoff parameters). Table 5 shows the specific information of the file. |
| **AvsB__pval.txt** | Table containing the FDR or p-adjust values of all genes |
| **AvsB__logFC.txt** | Table containing the logFC values of all genes |
| **AvsB__Abundances.txt** | Table containing the raw and normalized counts of all samples |
| **AvsB__intersect.txt** | Table that contains the results of differential expression of those genes that are at the intersection of the DE genes obtained by all the different selected methods. Table 5 shows the specific information of the file. |

# 6 Result integration

Once the results of the differential expression analyses has been obtained (by the different selected methods), these results are compared, by mean of [Venn digram], Upset plot and Correlograms plots, in order to see how the different methods agree on the final list of differential expressed genes. Heatmaps are also plotted to visualize the expression values across the individual samples. Moreover, several file text are generated.

Table 5: Output files table

| *Header* | Description |
|---|---|
| **edgeR** | |
| *logFC* | The log fold-change between conditions being tested |
| *logCPM* | Average log2-counts per million, the average taken over all libraries |
| *PValue* | p value for the statistical significance of the change |
| *FDR* | p value adjusted for multiple testing with the Benjamini-Hochberg procedure[Reference], which controls false discovery rate (FDR) |
| **limma** | |
| *logFC* | The log fold-change between conditions being tested |
| *AveExpr* | Average log2 expression |
| *t* | the t-statistic used to assess differential expression |
| *P.Value* | The p-value for differential expression; this value is not adjusted for multiple testing |
| *adj.P.Val* | The p-value adjusted for multiple testing |
| *B* | The B-statistic is the log-odds that the gene is differentially expressed |
| **DESeq2** | |
| *baseMean* | Mean normalised counts, averaged over all samples from the two conditions |
| *log2FoldChange* | The log fold-change between conditions being tested |
| *lfcSE* | Standard errors of logarithm fold change |
| *stat* | test statistics |
| *pvalue* | p value for the statistical significance of this change |
| *padj* | p value adjusted for multiple testing with the Benjamini-Hochberg procedure [reference] |
| **NOISeq** | |
| *Cond1_mean* | The mean of the biological replicates of Cond1 |
| *Cond2_mean* | The mean of the biological replicates of Cond2 |
| *theta* | Differential expression statistics |
| *prob* | Probability of differential expression |
| *log2FC* | The log fold-change between conditions being tested |
| **All methods** | |
| *raw counts* | Raw counts for all samples |
| *normalized counts* | Normalized counts for all samples |
| *Regulation* | gene regulation, indicating how and in which condition the genes was expressed |

## 6.1 Integration output plots

### 6.1.1 Venn diagram

The Venn diagram shows the intersection of the differentially expressed genes reported by each of the selected methods (Figure 19). Further analysis could be considered only in the list of genes that were identified as differentials expressed by more than one method.

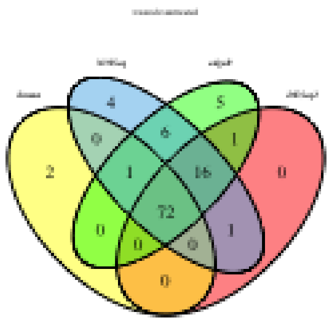


Figure 19: Venn diagram

### 6.1.2 Upset plot

The UpSet plot visualizes set intersections in a matrix layout. The matrix layout enables the effective representation of associated data, such as the number of elements in the aggregates and intersections(Lex A 2014). This graph, like the Venn diagrams, shows the intersection of the differentially expressed genes reported by each of the selected methods (Figure 20).

### 6.1.3 Correlograms

Correlograms (Wei and Simko 2017) help us visualize the data in correlation matrices. This scheme shows both the sign and magnitude of the correlation value. In the integration results step, three different correlograms are generated: [p-values correlogram], [logFC corelogram] and Abundance correlograms. In the following sections each type of correlograms is explained. Figure 21, shows an example.

#### 6.1.3.1 pvalues correlogram

The pvalues corrlogram represents the correlation of padjust / FDR values that each method calculated for differentially expressed genes. The closer the correlation value is to 1, the more similar the results are.

#### 6.1.3.2 logFC correlogram

The logFC corrlogram represents the correlation of logFC values that each method calculated for differentially expressed genes.

#### 6.1.3.3 Abundance correlograms

Represents the correlation of the normalized values of each sample performed by each method. A correlogram is plotted for each condition.

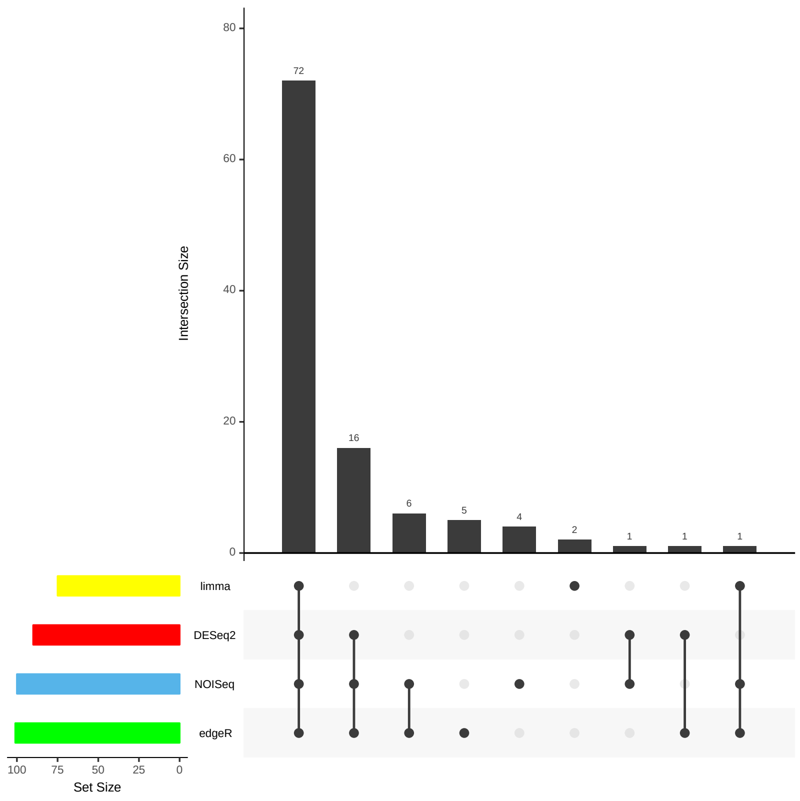


Figure 20: Upset plot

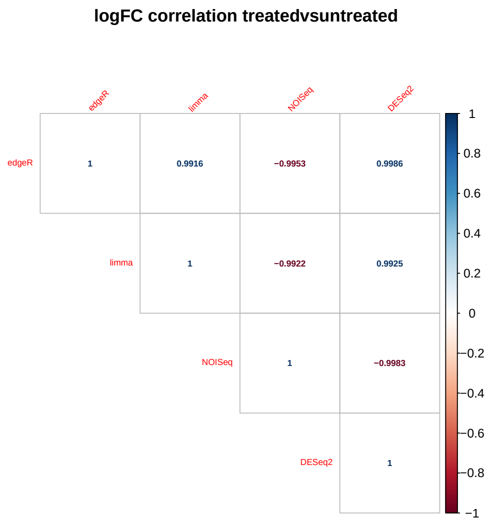


Figure 21: Correlograms

### 6.1.4 Heatmaps

Heatmap is a common means to visualize gene expression data. In the heatmaps the data is displayed in a grid where each row represents a gene and each column represents a sample. The colour and intensity of the boxes is used to represent changes of gene expression. There are numerous functions for generating heatmaps in R; IDEAMEX uses a function called `heatmap()` of the Bioconductor `ComplexHeatmap` package (Gu Z 2016), to perform this task.

IDEAMEX generates two heatmaps for each pairwise comparison. The first one is performed with the intersection of the differentially expressed genes reported by each of the selected methods. And the second one is carried out with union of the differentially expressed genes reported by all the selected methods. It is important to mention that if the number of genes to be plotted exceeds 200, only 200 will be plotted.

## 6.2 Integration output text files

In the process of integration results, different files are obtained. These files contain information related to the differentially expressed genes found at the intersection of all methods. In table 6 shows the description of the files.

Table 6: Output files table

| **Files** | Description |
|---|---|
| **AvsB__table.txt** | File of binary values, which indicate for each gene, the methods that report it as differentially expressed. In the last column of the file, a description of the gene regulation can be found, where is indicated how and in which condition the genes was expressed. |
| **AvB__IntersectSummary.txt** | Contains the summary of the number of DE genes in all possible logical relationships between the different methods |
| **AvsB__Intesrsect__TOP__IDs.txt** | Table that contains the ID of those genes that are at the intersection of the DE genes obtained by all the different selected methods. |
| **AvsB__logFCTable.txt** | Table containing the logFC values of the DE genes reported for all methods |
| **AvsB__AbundanceTable.txt** | Table containing the raw and normalized counts of all samples of the DE genes reported for all methods |
| **AvsB__PvalTable.txt** | Table containing the padjust/FDR values of the DE genes reported for all methods |

# 7 Case of studies

This section provides the detailed steps for differential expression analysis in three different case studies, considering a) Pairwise comparisons between two or more groups. b) RNA-Seq with batch effect.

## 7.1 Comparison between two groups

The analysis starts with the upload of a gene abundance count table per gene like the one shown in the following image:

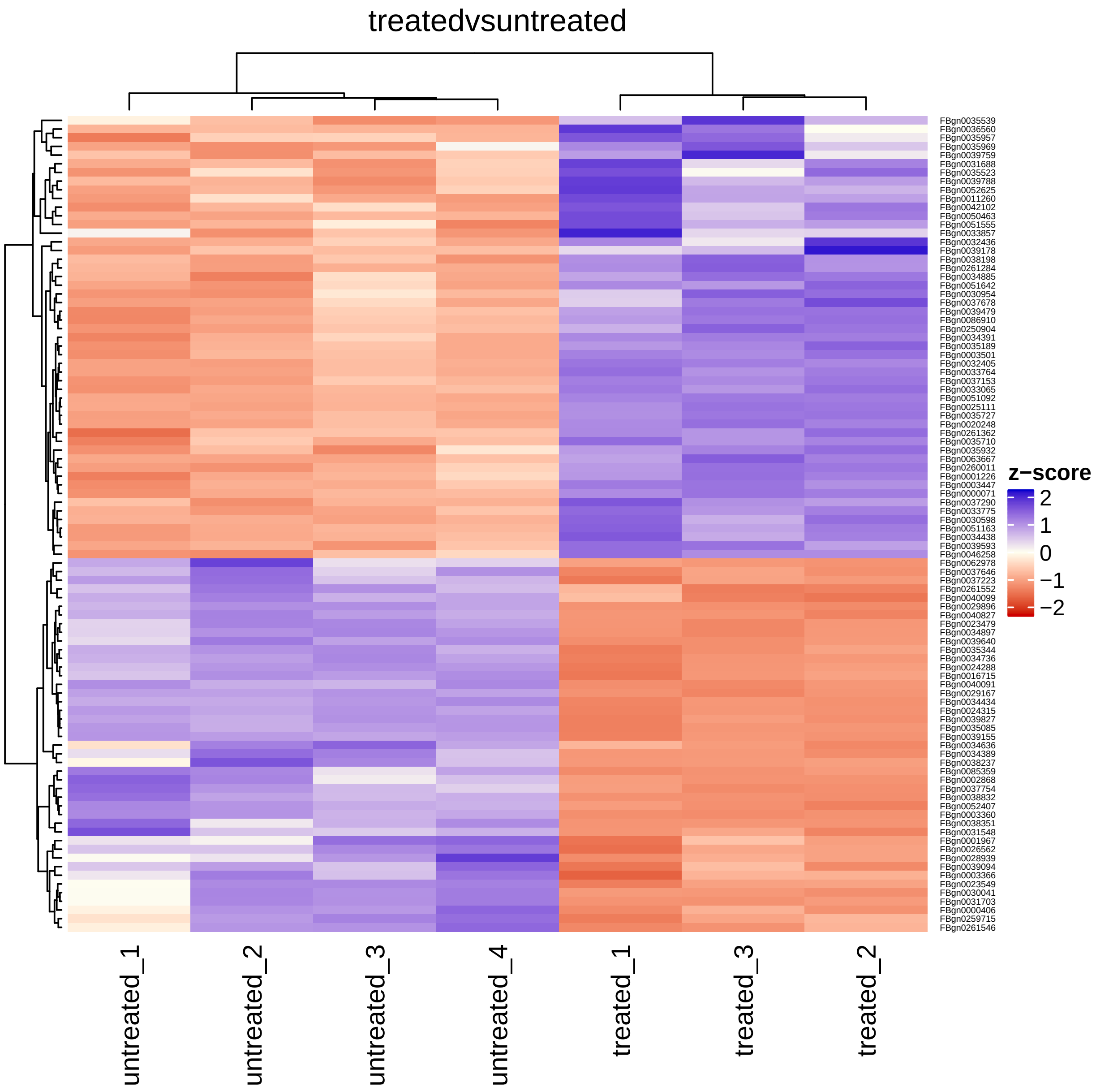


Figure 22: Heatmap. Visualization of gene expression data

Table 7: Raw count table example file

| | untreated_1 | untreated_2 | untreated_3 | untreated_4 | treated_1 | treated_2 | treated_3 |
|---|---|---|---|---|---|---|---|
| **FBgn0000003** | 0 | 0 | 0 | 0 | 0 | 0 | 1 |
| **FBgn0000008** | 92 | 161 | 76 | 70 | 140 | 88 | 70 |
| **FBgn0000014** | 5 | 1 | 0 | 0 | 4 | 0 | 0 |
| **FBgn0000015** | 0 | 2 | 1 | 2 | 1 | 0 | 0 |
| **FBgn0000017** | 4664 | 8714 | 3564 | 3150 | 6205 | 3072 | 3334 |
| **FBgn0000018** | 583 | 761 | 245 | 310 | 722 | 299 | 308 |

Requisites for table format (explained in Section 3.1): 1. Must be in simple text format with values separated by tabulators 2. In the first column, gene names or IDs must be unique 3. The names in the first column must have no spaces or special symbols 4. All rows must have the same number of columns. 5. The first row must start with a tabulator.

In the page http://www.uusmb.unam.mx/ideamex/ the user must provide a valid email address and I load the table file. In this example, we'll use the DrosofilaCount.txt file that can be found in the same box. Please, download the file and then select it. Then press the submit button:

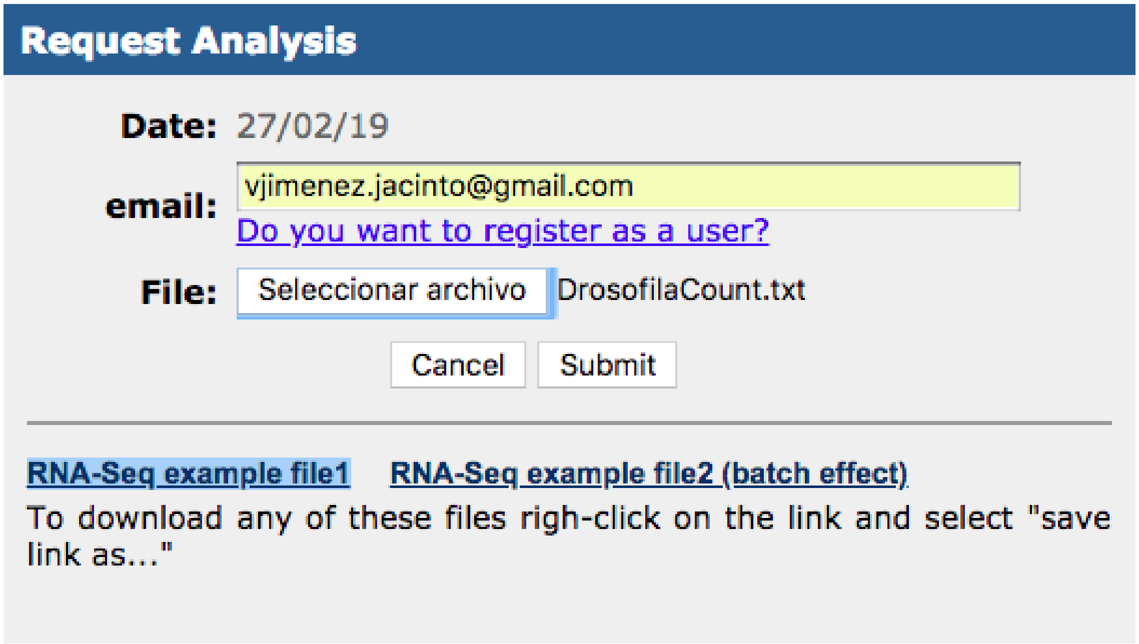


To perform the preliminary analysis of data, all differential expression methods, and data integration of results, please select the option "ALL" and press the "Continue" button:

In the next page, IDEAMEX reports at the top of grey box. The selected methods that will be applied to the input datatable of counts, and will displays the names that I found in the header line of the file. In this box, the names of the conditions or experiments can be edited as explained in Section 3.1.1 of this manual. In this particular example, samples for each condition were prepared in the same batch. Therefore, there is no need to provide any batch information. Please, press the "Continue" button to select which comparison combinations will be performed.

IDEAMEX will show a box with a matrix with all the condition names. Here, it can be defined the comparisons to be performed. In this example, there are only two conditions: untreated and treated. However, is important to define which one will be the "reference". In this image below, untreated will be selected as the reference conditions for the expression directionality (UP or DOWN). Please, check the "TOP" option to generate a file with results that are below the threshold values (False Discovery Rate; p-adjusted value; logFC and

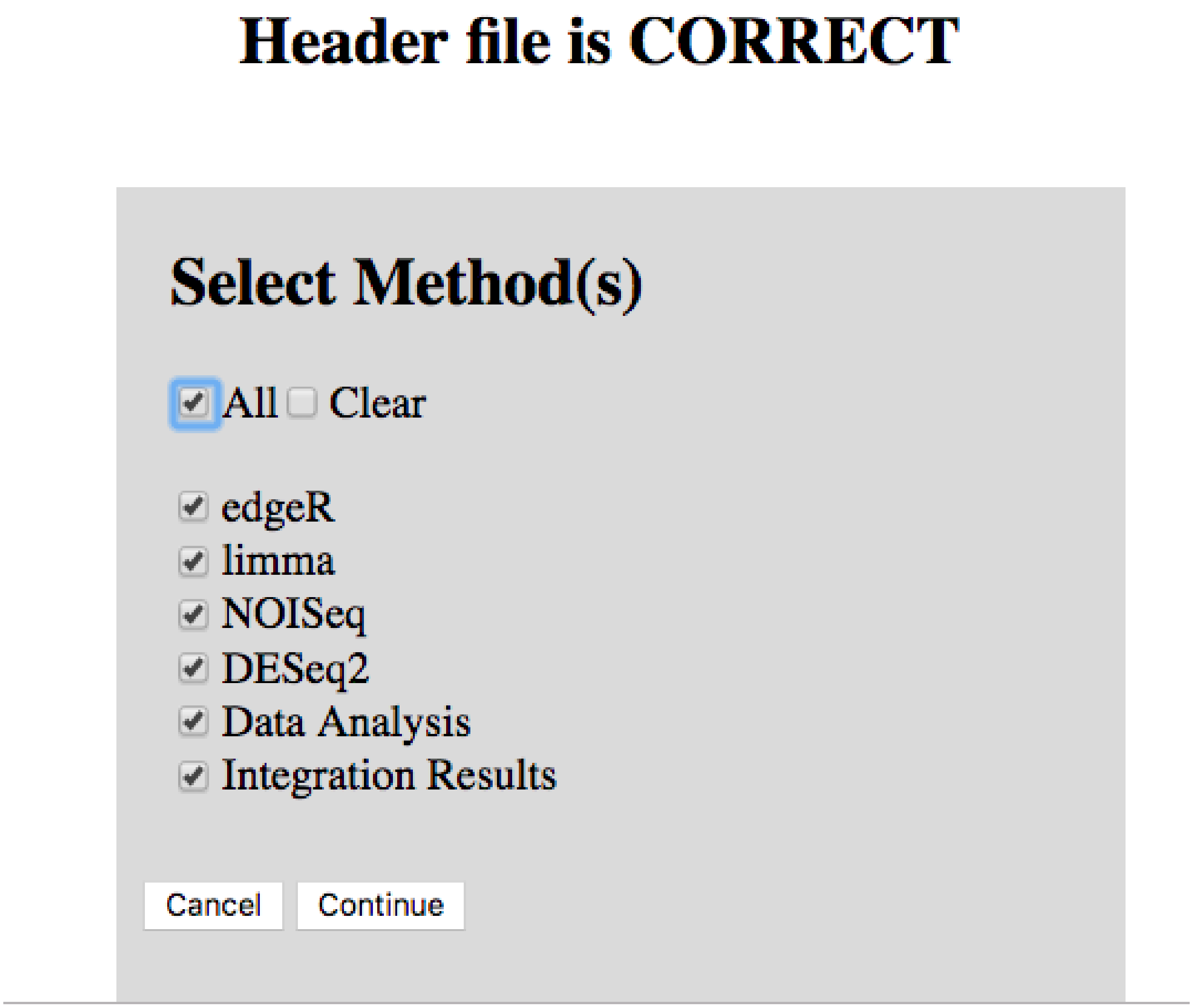


Figure 23: SelectMethod. Select ALL methods for analysis

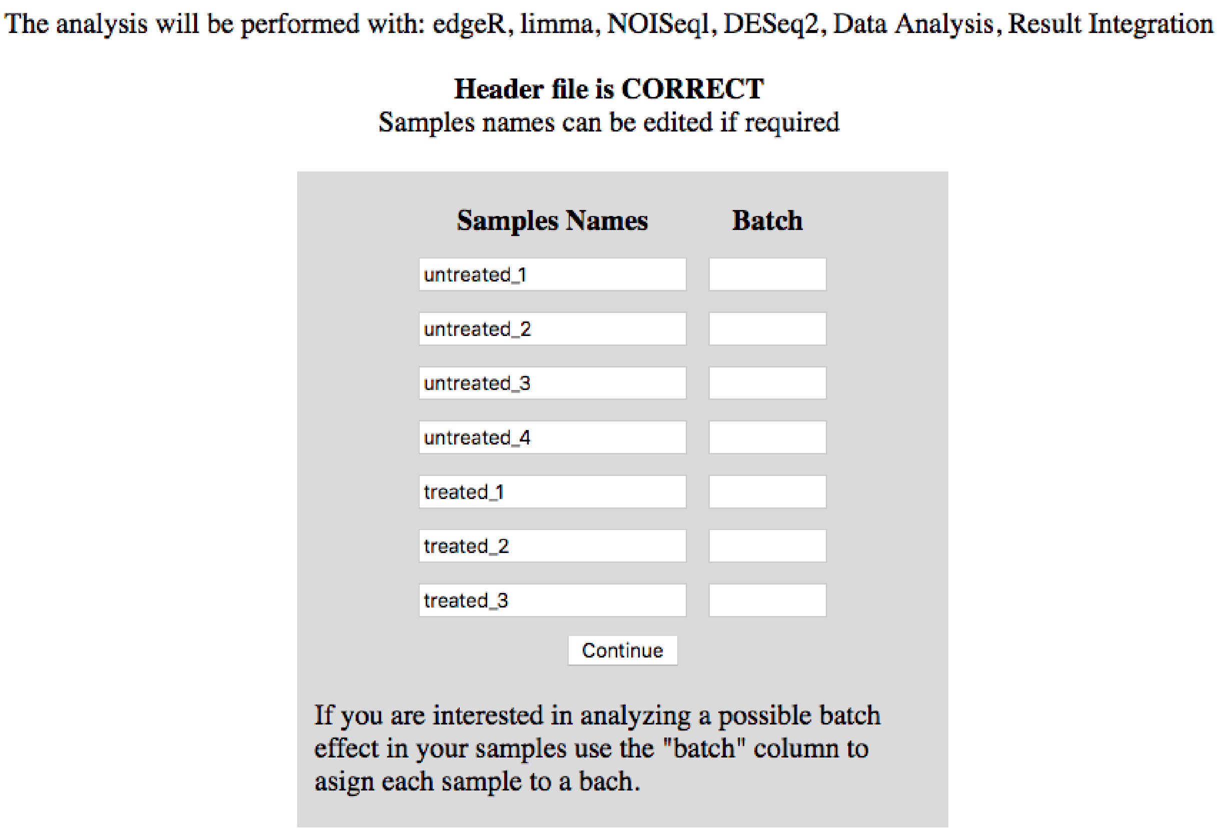


Figure 24: SelectMethod. Select ALL methods for analysis

CPM). It is advisable to use a CPM (Count Per Million) value to discard from the analysis those genes with very low counts or abundance. Is recommended to use the default threshold values.

NOTE: If the analysis has more than two conditions, all condition combinations can be selected. Bear in mind that usually, one of the conditions is used as reference and some comparisons won't have any sense, especially in a time line expression analysis, or in a wild type vs mutants experiments.

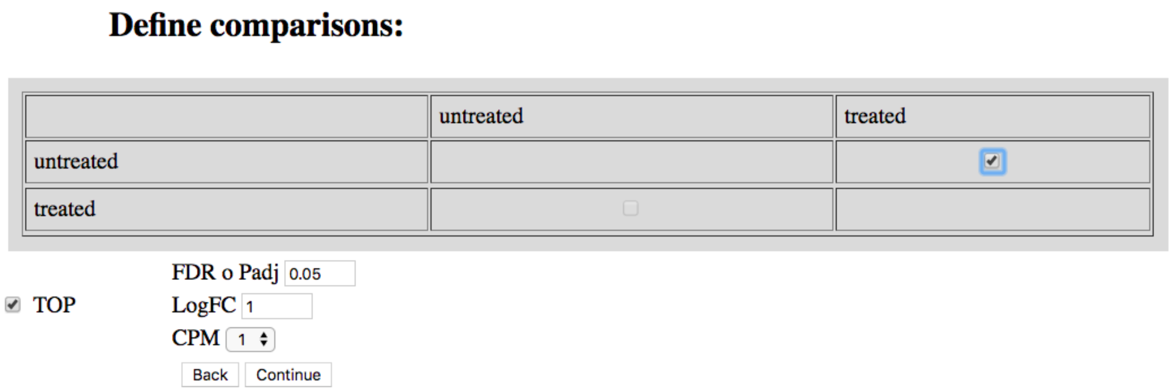


Figure 25: DefineComparation.Defined the comparisons to be performed

After pressing the Continue button, it would take several minutes before IDEAMEX shows a page with a similar

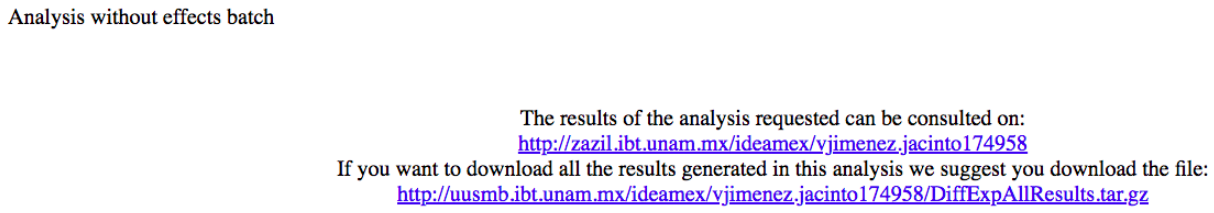


message:
The URL link can be used to load the results page. This same link will be sent to the registered email. The second URL can be used to retrieve a TAR file with all the results generated in the analysis.

When the results are ready, a screen similar to the following will be shown:

Please, navigate the results and analyze the results and plots that are fully described in Sections 4, 5 and 6 of this manual.

## 7.2 RNA-Seq with batch effect

Batch effect means that replicates from a given condition are prepared in a different time or by a different person, the samples can present an extra variability that could potentially affect the analysis. It mostly depends on how carefully people prepare the samples, although some batch effect is inevitable. Ideally, all samples for a given condition, should be all prepared together and by the same person to minimize possible batch effects.

In this case, we'll use the second example file that can be found in the "Request Analysis" box. Please, download the (arab.txt) file locally and then select it. The file will contain data from 2 conditions with 3 replicates each. However, information regarding samples "mock_3" and "hrcc_1", indicates that both samples were prepared in the same batch, while mock_2 and hrcc_2 were prepared in a second batch. Finally, samples mock_1 and hrcc_3 were prepared in a third batch. This introduces a batch effect that can hinder the analysis.

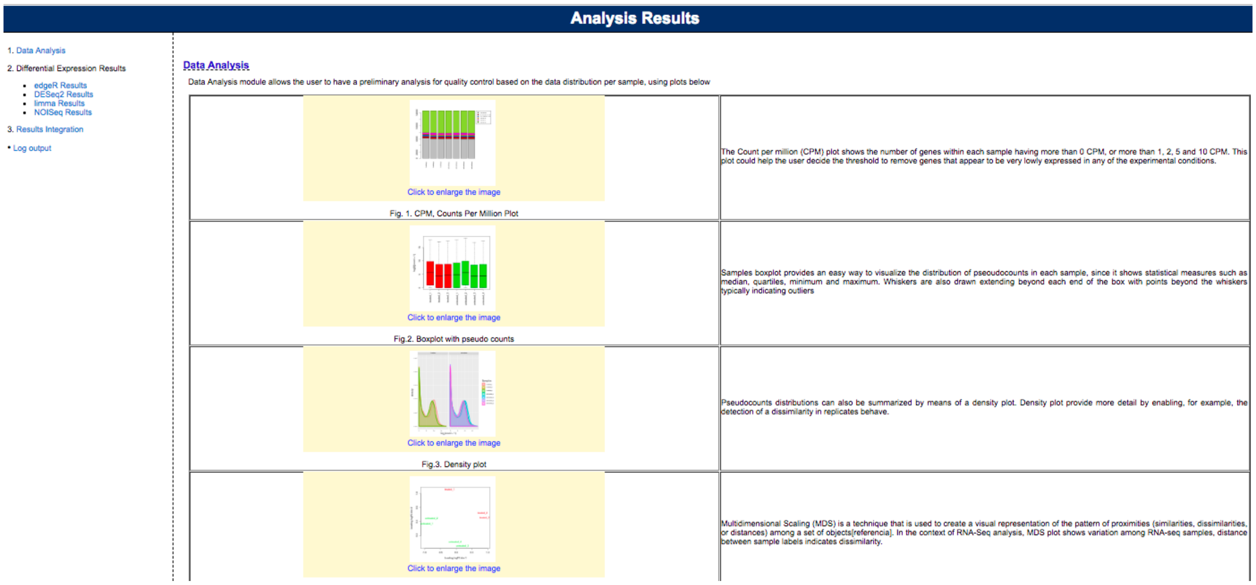


Figure 26: LinkResultsDroshopila

NOTE: Usually, information regarding the sample preparation is not available and the batch effect could be inferred from the PCA and MDS plots. However, this is not trivial and the information from the wet lab will be always more useful.

Table 8: Raw count table example file with batch effect

| | mock_3 | mock_2 | mock_1 | hrcc_1 | hrcc_2 | hrcc_3 |
|---|---|---|---|---|---|---|
| **AT1G01010** | 35 | 77 | 40 | 46 | 64 | 60 |
| **AT1G01020** | 43 | 45 | 32 | 43 | 39 | 49 |
| **AT1G01030** | 16 | 24 | 26 | 27 | 35 | 20 |
| **AT1G01040** | 72 | 43 | 64 | 66 | 25 | 90 |
| **AT1G01050** | 49 | 78 | 90 | 67 | 45 | 60 |
| **AT1G01060** | 0 | 15 | 2 | 0 | 21 | 8 |

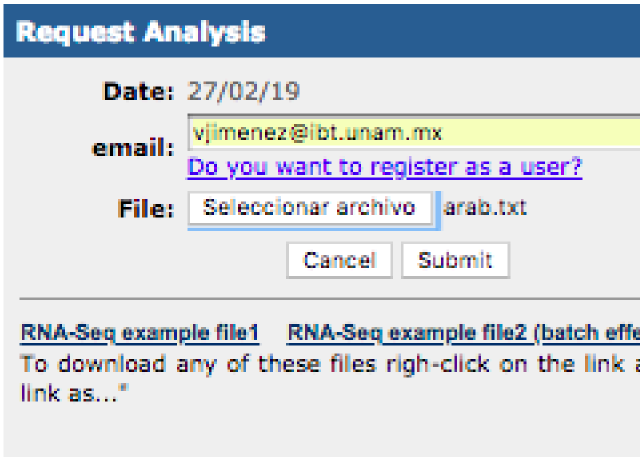


Please, after selecting the example file, press the submit button:

IDEAMEX will show the next page after validating the input file where the “Header file is CORRECT” message should be shown. Please, check the box the option “ALL” select all methods and press the “Continue” button

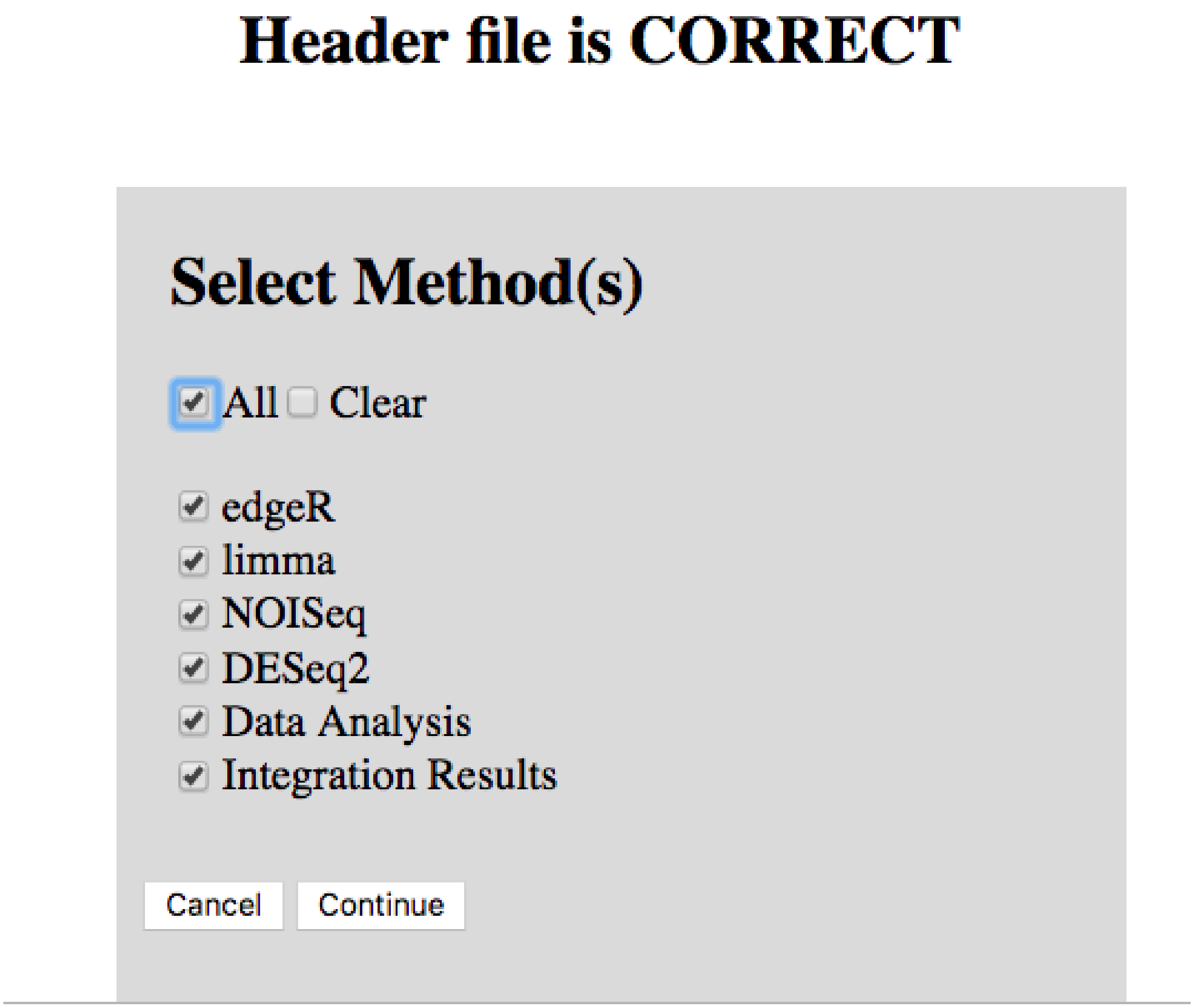


Figure 27: SelectMethod. Select ALL methods for analysis

Please, in this example specify in the batch column, the information provided for samples prepared in three different batches. Remember that samples mock3 and hrcc1 belong to the same batch. It is important to mention that when using the batch column, is necessary to define at least one pair of samples that belong to the same batch without being from the same condition. Once a batch is defined, you have to assign batch numbers to the rest of the samples.

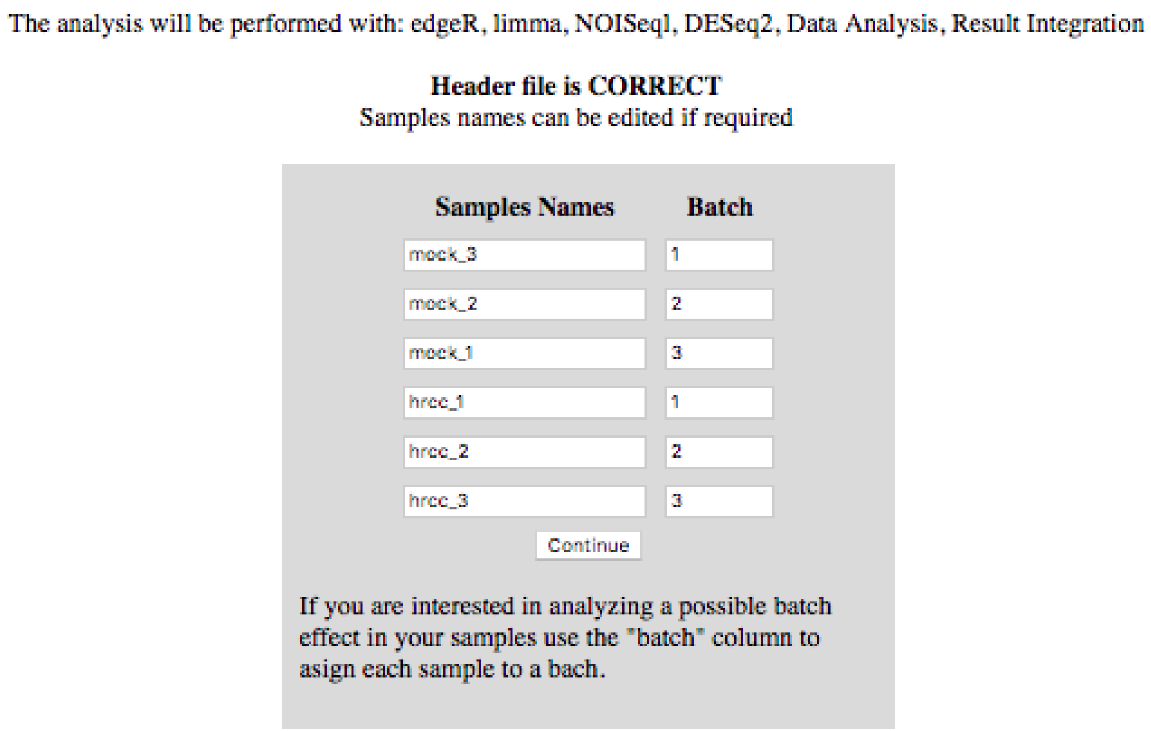


By pressing the “Continue” button IDEAMEX will display the matrix with the conditions, tissues or treatments found in the header information. Please, choose the conditions to be compared and threshold values as described before.

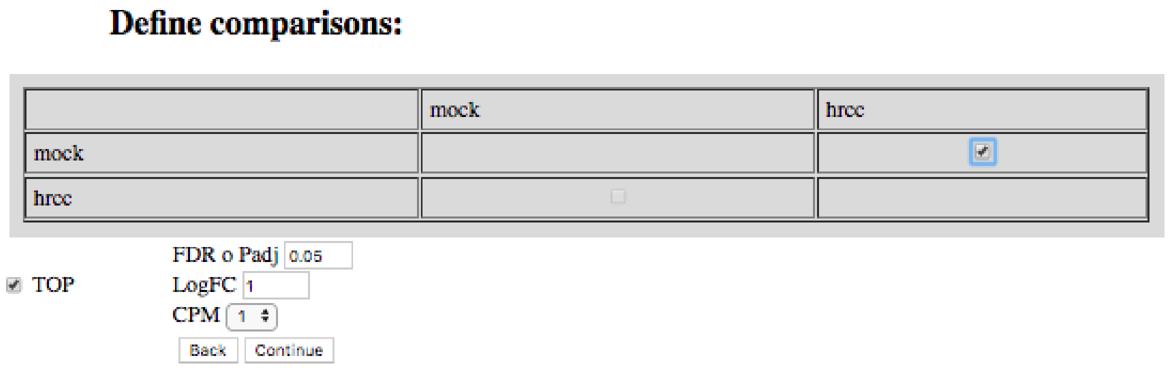


Finally, IDEAMEX may take a few minutes to respond with the path where the results will be displayed. This same information is sent to the email provided at the beginning of the analysis.

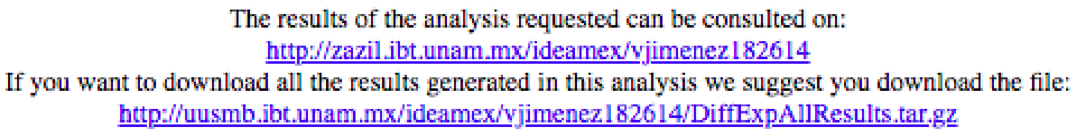


Figure 28: ResultAra. Results with batch effect

Since batches were defined, differential expression methods with batch error correction will consider the extra variability between replicates from a given condition. If you analyze the PCA and MDS plots, you will notice that samples mock_2 and hrcc2 present a higher dispersion that separate them from their condition group but are not clustered with samples from a different condition. This suggest a batch effect.

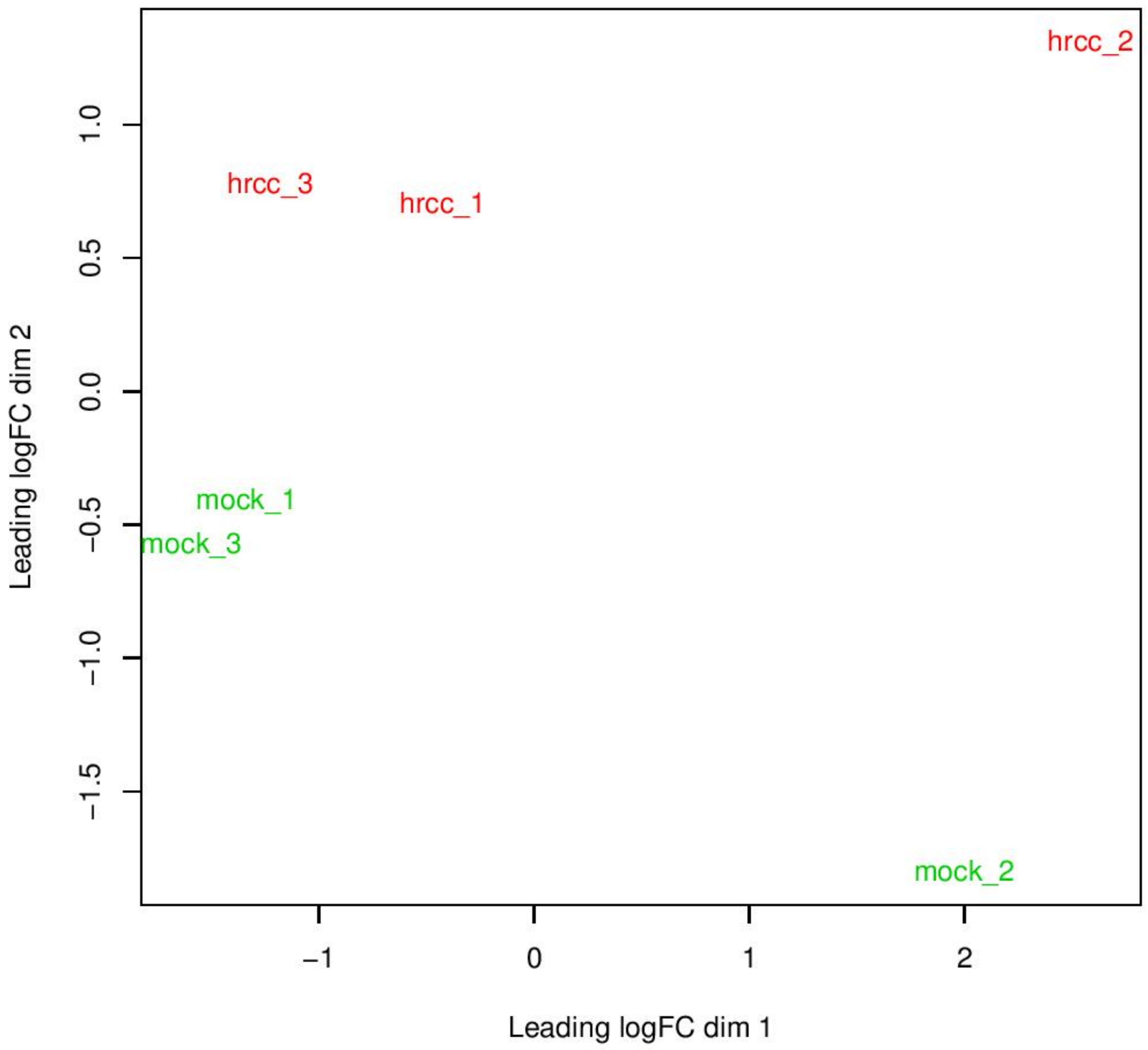


Figure 29: BeforeBatch. MDSplot with Batch example

However, since batches were defined, that will turn on the batch effect error correction in all methods, (except ...) which will be reflected in the results. The batch effect correction will improve the results as observed in the following image

Correction the batch effect error will filter the "noise" generated by the extra variability originated from the sample preparation. Usually, this will increase the sensitivity of the methods and the results intersection would be larger.

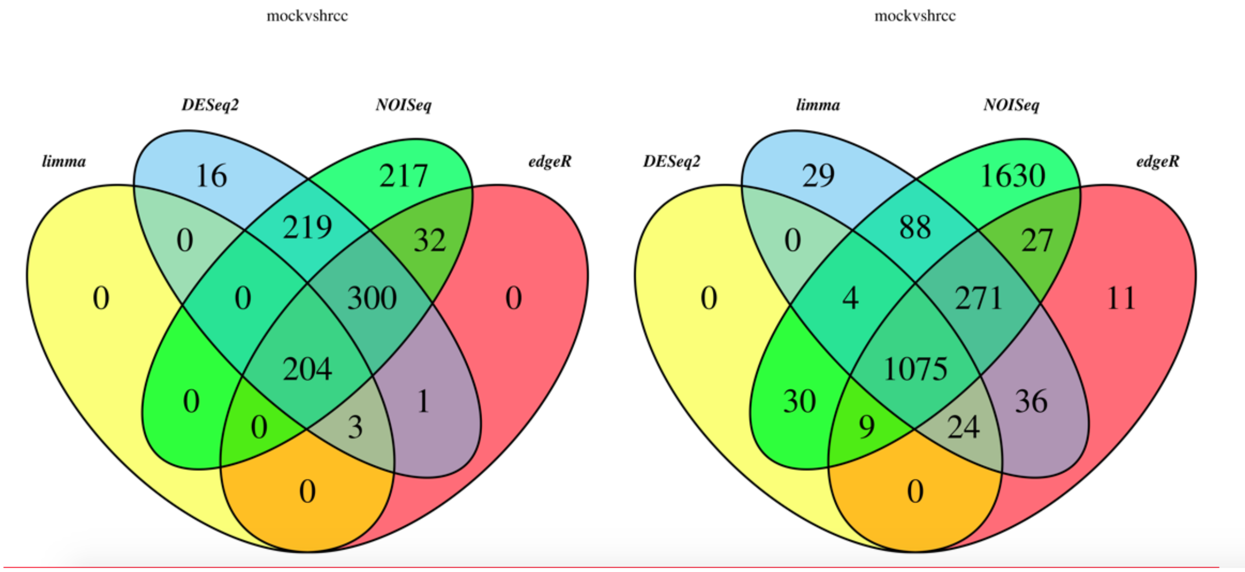


Figure 30: BatchError. A) Before batch effect error B correction) After batch error effect correction

# References


Anders S, Chen Y, McCarthy DJ. 2013. "Count-Based Differential Expression Analysis of Rna Sequencing Data Using R and Bioconductor." *Nature Protocols* 8: 1765, 1786.

Brooks AN, Duff MO, Yang L. 2010. "Conservation of an Rna Regulatory Map Between Drosophila and Mammals." *Genome Research* 21 (12): 193, 202.

Cumbie JS, Di Y, Kimbrel JA. 2011. "Gene-Counter: A Computational Pipeline for the Analysis of Rna-Seq Data for Gene Expression Differences." *PLoS ONE* 6: e25279. doi:10.1371/journal.pone.0025279.

Di Y, Cumbie JS, Schafer DW. 2014. "Negative Binomial Model for Rna-Sequencing Data." https://cran.r-project.org/web/packages/NBPSeq/index.html.

Goh WWW, Wong L, Wang W. 2017. "Why Batch Effects Matter in Omics Data, and How to Avoid Them." *Trends in Biotechnology* 35 (6): 498–507. doi:doi.org/10.1016/j.tibtech.2017.02.012.

Gonzalez, I. 2014. *Tutorial. Statistical Analysis of Rna-Seq Data.* Plateforme Bioinformatique INRA Toulouse, France.

Gu Z, Schlesner M, Eils R. 2016. "Complex Heatmaps Reveal Patterns and Correlations in Multidimensional Genomic Data." *Bioinformatics* 32 (18): 2847, 2849. doi:10.1093/bioinformatics/btw313.

Huber W, Gentleman R, Carey VJ. 2015. "Orchestrating High-Throughput Genomic Analysis with Bioconductor." *Nature Methods* 12: 115, 121. doi:10.1038/nmeth.3252.

Lex A, Strobelt H, Gehlenborg N. 2014. "UpSet: Visualization of Intersecting Sets." *IEEE Transactions on Visualization and Computer Graphics (InfoVis '14)* 20 (12): 1983, 1992. doi:10.1109/TVCG.2014.2346248.

Love MI, Anders S, Huber W. 2014. "Moderated Estimation of Fold Change and Dispersion for Rna-Seq Data with Deseq2." *Genome Biology* 15 (550): 1, 21. doi:10.1186/s13059-014-0550-8.

Ritchie ME, Wu D, Phipson B. 2015. "Limma Powers Differential Expression Analyses for Rna-Sequencing and Microarray Studies." *Nucleic Acids Research* 43 (7): e47.

Robinson MD, Smyth GK, McCarthy DJ. 2010. "EdgeR: A Bioconductor Package for Differential Expression

Analysis of Digital Gene Expression Data.” *Bioinformatics* 26 (1): 139, 140.

Tarazona S, Dopazo J, Garcia-Alcalde F. 2011. “Differential Expression in Rna-Seq: A Matter of Depth.” *Genome Research* 21 (12): 4436.

Tarazona S, Turra D, Furio-Tari P. 2015. “Data Quality Aware Analysis of Differential Expression in Rna-Seq with Noiseq R/Bioc Package.” *Nucleic Acids Research* 43 (21): e140.

Wei, Taiyun, and Viliam Simko. 2017. *R Package “Corrplot”: Visualization of a Correlation Matrix.* https://github.com/taiyun/corrplot.
